# Preparing for a future COVID-19 wave: insights and limitations from a data-driven evaluation of non-pharmaceutical interventions in Germany

**DOI:** 10.1038/s41598-020-76244-6

**Published:** 2020-11-18

**Authors:** Ashwin Aravindakshan, Jörn Boehnke, Ehsan Gholami, Ashutosh Nayak

**Affiliations:** University of California, Davis, Davis, CA USA

**Keywords:** Health policy, Public health

## Abstract

To contain the COVID-19 pandemic, governments introduced strict Non-Pharmaceutical Interventions (NPI) that restricted movement, public gatherings, national and international travel, and shut down large parts of the economy. Yet, the impact of the enforcement and subsequent loosening of these policies on the spread of COVID-19 is not well understood. Accordingly, we measure the impact of NPIs on mitigating disease spread by exploiting the spatio-temporal variations in policy measures across the 16 states of Germany. While this quasi-experiment does not allow for causal identification, each policy’s effect on reducing disease spread provides meaningful insights. We adapt the Susceptible–Exposed–Infected–Recovered model for disease propagation to include data on daily confirmed cases, interstate movement, and social distancing. By combining the model with measures of policy contributions on mobility reduction, we forecast scenarios for relaxing various types of NPIs. Our model finds that in Germany policies that mandated contact restrictions (e.g., movement in public space limited to two persons or people co-living), closure of educational institutions (e.g., schools), and retail outlet closures are associated with the sharpest drops in movement within and across states. Contact restrictions appear to be most effective at lowering COVID-19 cases, while border closures appear to have only minimal effects at mitigating the spread of the disease, even though cross-border travel might have played a role in seeding the disease in the population. We believe that a deeper understanding of the policy effects on mitigating the spread of COVID-19 allows a more accurate forecast of disease spread when NPIs are partially loosened and gives policymakers better data for making informed decisions.

## Introduction

In response to the COVID-19 pandemic, governments around the world implemented varying degrees of non-pharmaceutical interventions (NPIs) to control the spread of the disease^1–5^. These policies severely restricted movement, public gatherings, national and international travel, and shut down large parts of the economy including schools and non-essential businesses. Multiple studies have investigated the role that these lockdowns played in delaying the spread and reducing the severity of the pandemic^6,7–14^. The lockdowns also created tremendous hardships for individuals and businesses^15–17^. As the spread of COVID-19 decelerated across countries, governments began relaxing the NPIs to help balance the need for economic security against the risk of growing infection numbers^18^. Nevertheless, beyond the knowledge of the possibility of a future wave^19^, there is still limited understanding of the association of loosening different policies with changes in mobility that eventually relate to the spread of the disease.

In this study, we explore this association by estimating each NPI’s connection to social mobility and the resulting disease mitigation. The proposed methodology permits the forecasting of disease spread under different policy scenarios of implementation and relaxation by associating a policy with a change in mobility. The presented model allows policymakers to forecast the impacts of removing different types of restrictions on mobility and disease mitigation.

Initial analysis of the impact of policy restrictions in China suggests that NPIs that significantly affected human mobility (e.g., household quarantine) reduced the spread of the disease^20,21^, even more than restrictions that limited national and international travel^22^. Additionally, simulations of NPIs in Wuhan^13^ show that maintaining restrictions helped delay the epidemic peak. The results also suggest that an early end to such interventions leads to an earlier secondary peak, which can be flattened by relaxing the social mixing^13^. To the best of our knowledge no study quantifies the effects of the types and timings of the implementation and relaxation of government policy interventions in reducing mobility, and in turn decreasing the spread of COVID-19. Our estimates allow for projections of the impact of easing individual interventions on the spread of the disease. These projections act as aids for policymakers to determine how lifting certain policies will change social mobility, and in turn affect the number of new COVID-19 cases.

Using data from the 16 states of Germany, we explore the effectiveness of different NPIs in reducing social mobility, and in turn affecting the spread of the disease. Because German states enforced and relaxed policies to varying degrees at different points in time, the variations in implementation allow us to capture the incremental effectiveness of these policies at reducing social mobility in the general population. It is important to note that we cannot identify each policy’s causal effect on reducing disease spread. While each German state implemented their own COVID-19 interventions, the data do not offer sufficient variation in the sequencing of policies to uniquely identify the effect of each intervention. However, the existing variation allows for a necessary understanding of the relative magnitude of each of the interventions and provides meaningful insights of each policy’s contribution in reducing disease spread.

To determine how policy enforcement impacted mobility and disease spread, we associate the type and timing of the policy intervention to actual social mobility as recorded in the data released by Google^23^. Next, using our predictions of social mobility based on the policy interventions, we predict the spread of COVID-19 by modifying the SEIR model presented in^20^ to include social distancing and other forms of mobility data (e.g., travel by air, bus, rail, and road). Finally, we project the impact of relaxing a policy on the number of new cases across Germany and compare how differences in start times for policy relaxations alter the cumulative number of expected cases over a 90-day time span.

Our findings suggest that not implementing social distancing in Germany is associated with a 24.6-fold (IQR: 20 to 29-fold) increase in cumulative infected case counts as of May 7, 2020. In other words, social distancing appears to reduce case counts by about 96% (IQR: 95–96.6%). We also found that policies were not equal in their effectiveness at reducing new cases. Compared to keeping the restrictions in place, the lifting of contact restrictions appear to be the most punitive with an associated 150% (IQR: 144–156%) increase in daily case numbers over a 90-day period. This is followed by the lifting educational facilities closure and the opening of retail outlets (46.1% IQR: 44.0–48.1%, and 33.9% IQR: 33.0–34.8%), respectively.

## Method

In this work, we build an epidemiology model (Susceptible–Exposed–Infectious–Recovered, SEIR) to understand the impact of Non-Pharmaceutical Interventions (NPIs) in the spread of COVID-19 across Germany. We postulate that introduction of NPIs led to increase in social distancing that helped in containing the spread of the virus. We use Community Movement as an indicator for social distancing. First, we build a SEIR model that includes social distancing as a parameter. Then we use a linear regression model to estimate the impact of different NPIs on social distancing. We include other covariates that could simultaneously impact social distancing (e.g. awareness, weather, and state, week, day of week fixed effects). We use the coefficients from the linear regression model to simulate different scenarios and quantify the impact of NPIs using the SEIR model. We first discuss the data used in this work, followed by methods and results from our analysis.

### COVID-19 case data

**Figure 1:**
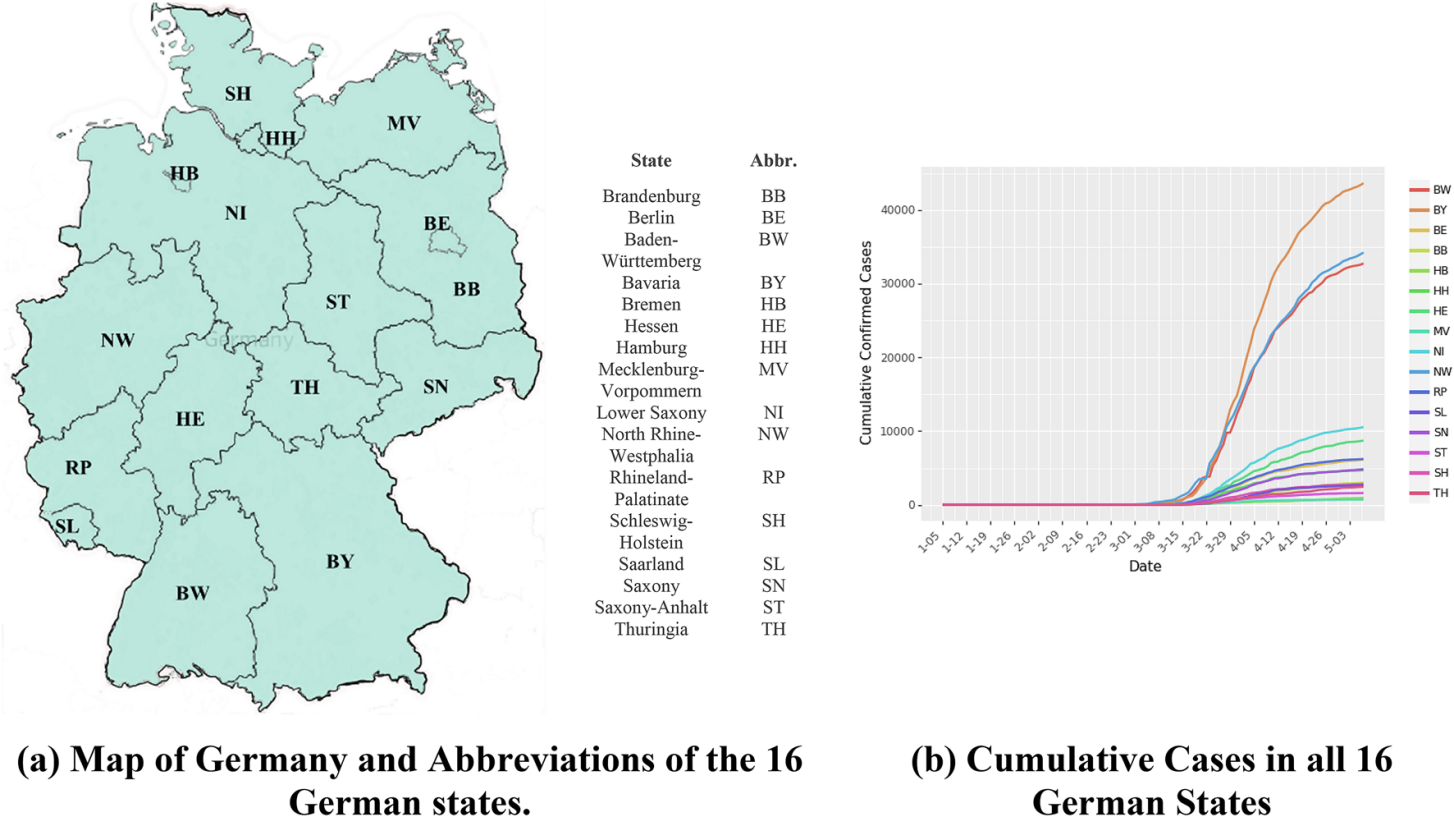
Map of Germany, Abbreviations of the 16 German states, and confirmed positive cases for COVID-19. Panel (**a**) shows a map of Germany and the abbreviations of the 16 German states (Figures generated using Tableau Software, Version 2020.2). Panel (**b**) shows the cumulative curves for the 16 states of Germany.

Interconnected air, land, and sea transportation networks led to the spread of COVID-19 from Wuhan, China to the rest of China, and eventually to most countries around the world^24,25^. To accurately model the spatial spread of the disease into Germany, we collected three types of daily mobility data: (1) daily air transportation data to capture the movement within and between Germany and 142 other countries; (2) daily ground transportation data between the nine countries that share borders with Germany; and (3) daily inter-state ground transportation. The daily COVID-19 case data were obtained from the Johns Hopkins Coronavirus Resource Center^26^ and Robert Koch Institute^27^ for all countries in our dataset as well as the 16 German states. Figure 1 introduces the states and the cumulative case numbers for all states.

### Government policies

**Figure 2:**
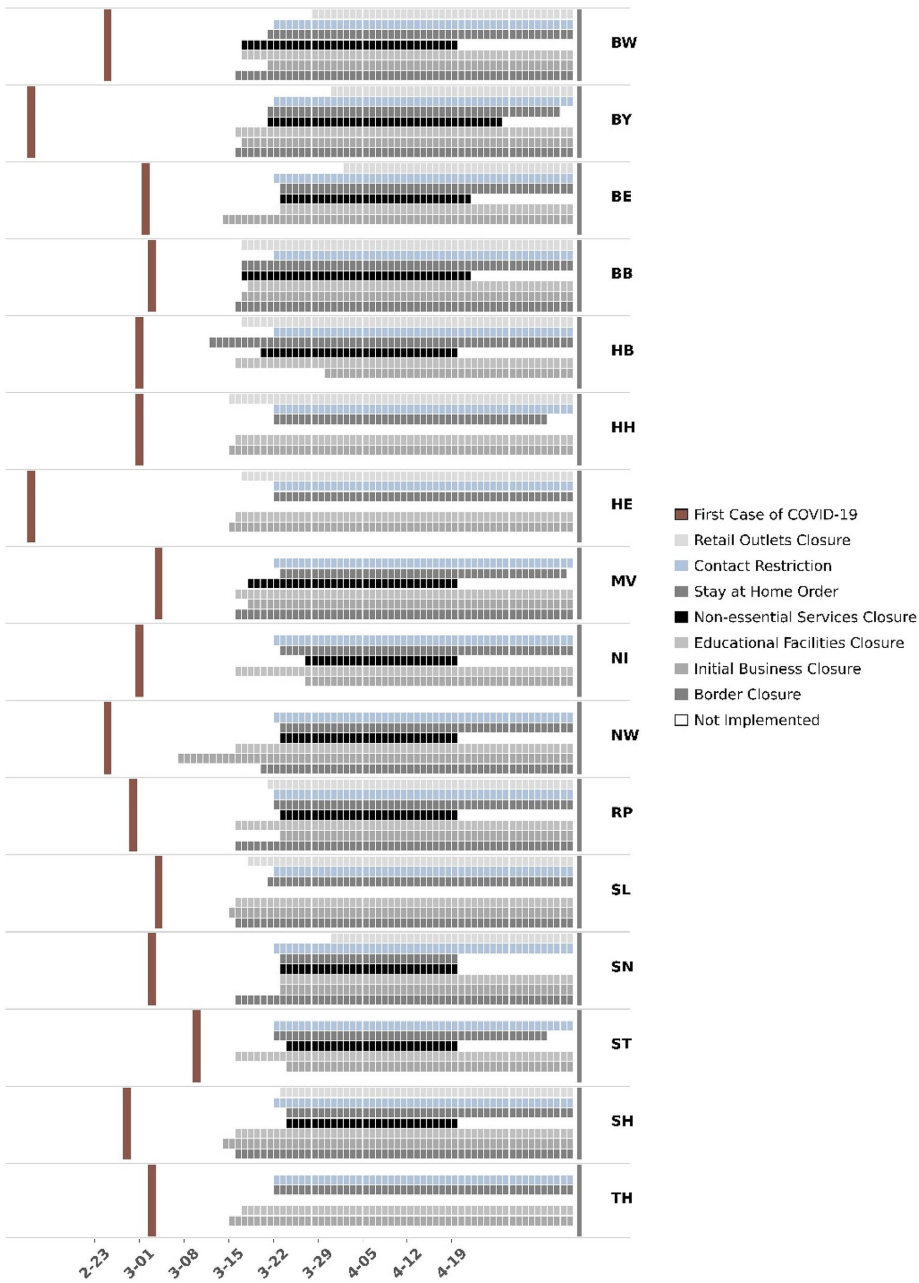
Timeline for implementation of NPIs across states. The policies are visualized in the same order from top to bottom for each state. These policies include, in this order, contact restrictions (movement in public space is limited to two persons or people co-living), initial business closures (e.g. restaurants), retail outlet closures, stay at home orders, non-essential business closures (e.g. trade shows), closure of educational institutes (e.g. schools and universities), and border closures (closing international borders). Border closures apply to 10 states sharing international borders. We use data from February 18, 2020 to May 7, 2020 in our study. Not every policy was implemented by each state as of April 20, 2020, and none of the implemented policies were relaxed until April 20. State governments started relaxing these policies after April 20, 2020.

To encourage and enforce physical distancing, governments across all 16 German states introduced a variety of NPIs at different points in time. Figure 2 provides an overview of the timeline. Data for these policies were collected from^28,29^, and Table S2 in the Supplementary Materials describes those policies. To understand the impact of each policy in containing the spread of the disease, we analyze (1) what would have happened if individuals did not reduce their mobility (i.e., if social distancing norms were never introduced), and (2) what would happen if a policy (*p*) is relaxed in the future. While the former can help governments in the early implementation of critical policies in case of future epidemics, the latter can help governments during current and future epidemics to decide which policies to relax first.

### Community mobility

**Figure 3:**
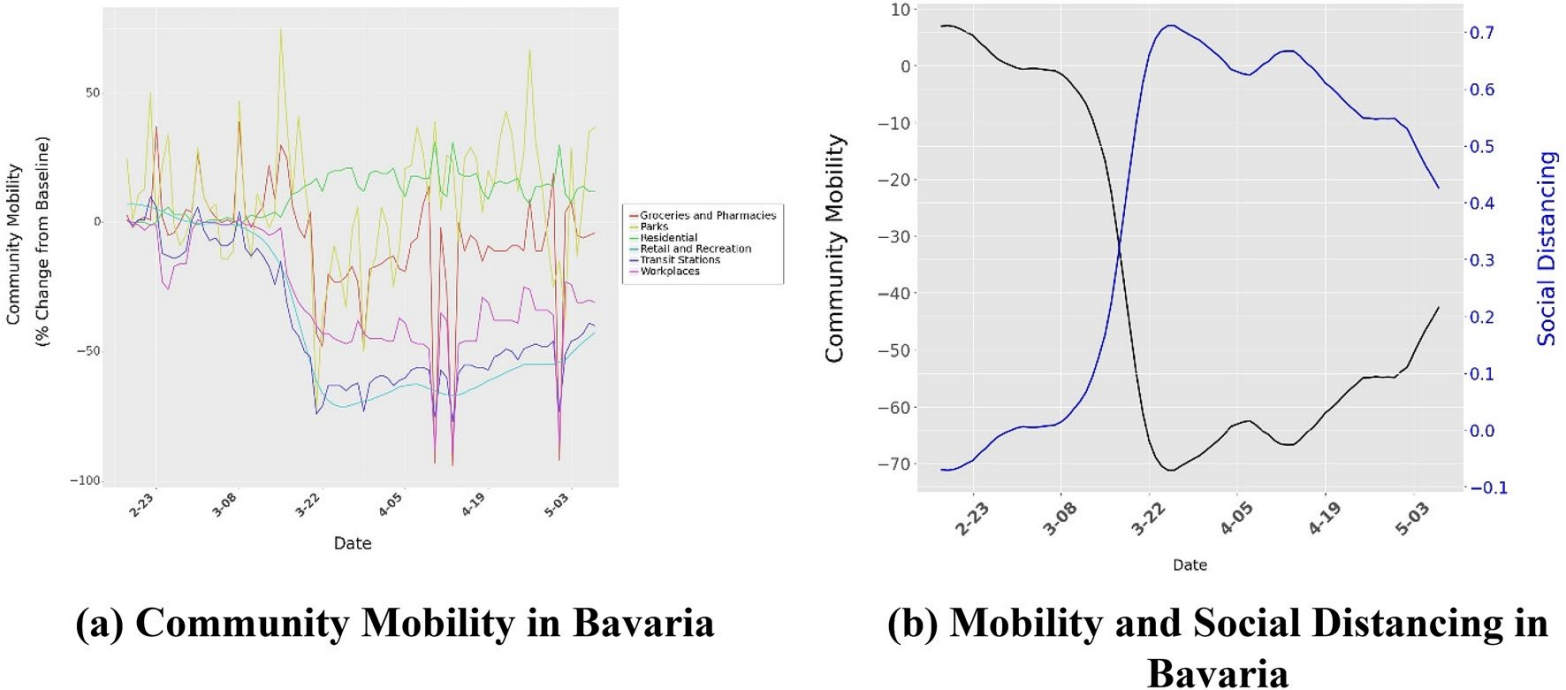
Community mobility and Social Distancing. Community mobility data charts the difference in foot traffic in different locations as compared to baseline from historical data. Panel (**a**) shows the movement trend across different categories of places for Bavaria. Similar movement trends for all the other states are shown in the Supplementary Material. Panel (**b**) shows the community mobility and social distancing in Bavaria.

Google’s COVID-19 Community Mobility Reports^23^ detail how movement trends change over time as public awareness increases and NPIs are introduced (Fig. 3). The report tracks movement trends over time by geography, across different categories such as retail and recreation, groceries and pharmacies, parks, transit stations, workplaces, and residential. We also consider Apple’s community movement reports^30^. As movement trends across locations are highly correlated (e.g., 0.92 between Apple’s “Driving” and Google’s “Retail and Recreation”), we only consider community mobility trends “Retail and Recreation” as a measure of social distancing (details in Supplement Section S3.1). We define social distancing as *sd*_*i*_ = *− C*_*i*_/100 where *C*_*i*_ is the community mobility trend in state *i*.

**Figure 4:**
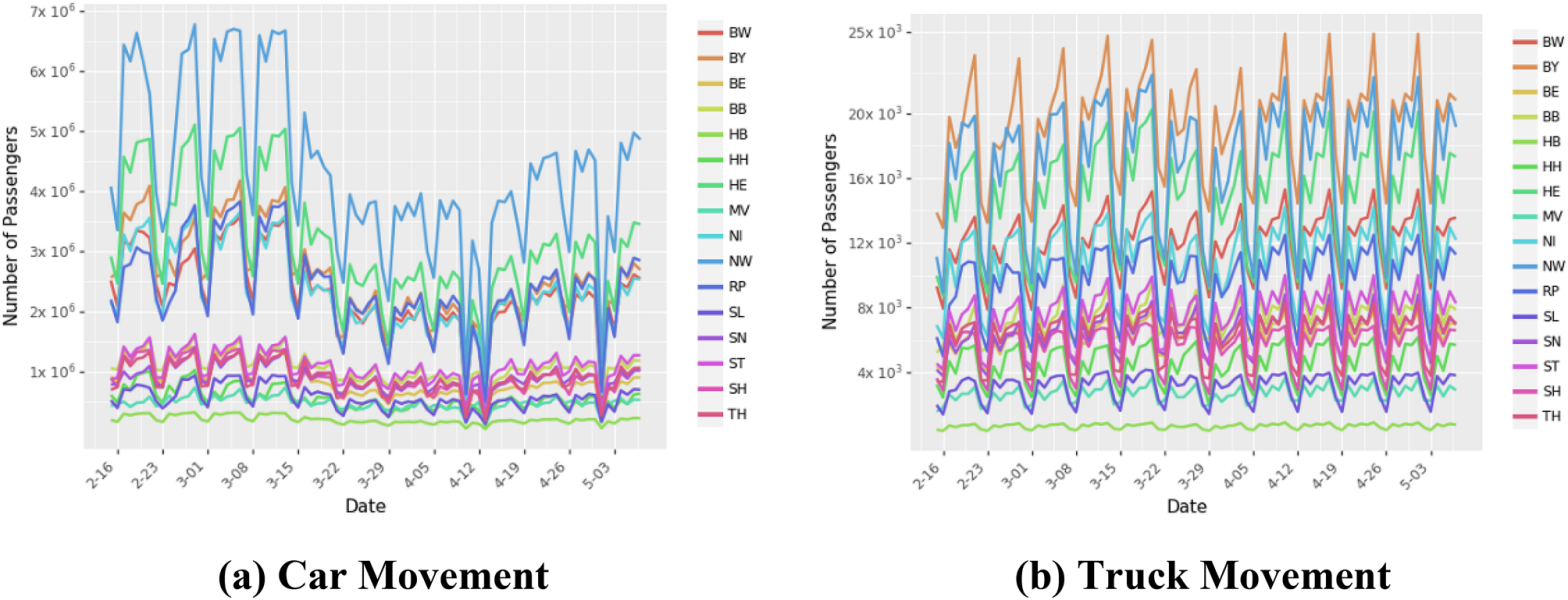
Ground transportation using cars and trucks in Germany. Panel (**a**) shows the number of passengers arriving in different states by car. Panel (**b**) shows the number of passengers arriving in different states. During the period of this study, there was no restriction on truck movement.

While the community mobility data provides information on changes in local movement, it does not provide information on inter-state movement. Ground transportation accounts for the vast majority of movement, with cars accounting for 85% of total ground transportation in Germany^31^. We collected detailed traffic data from Jan 1, 2013 to Dec 31, 2018 from the German Bundesanstalt für Straßenwesen (Federal Institute for Roadways). The dataset contains the hourly count of the number of vehicles crossing different checkpoints along highways across Germany. The institute used sensors to identify the type of vehicle, which we include in our analysis to estimate the number of individuals. We construct correction factors to extrapolate hourly traffic for Jan 1, 2020 to April 30, 2020 (details in Supplementary Material), and the model includes public holidays, day of the week, and state population as control variables. According to a survey, 61% of people in Germany use cars to commute to their workplace^32^. So, to control for changes in car movement during the period of the study, we adjust the predicted daily car movement between states using Google’s community mobility data for workplaces (Fig. 4).

**Figure 5:**
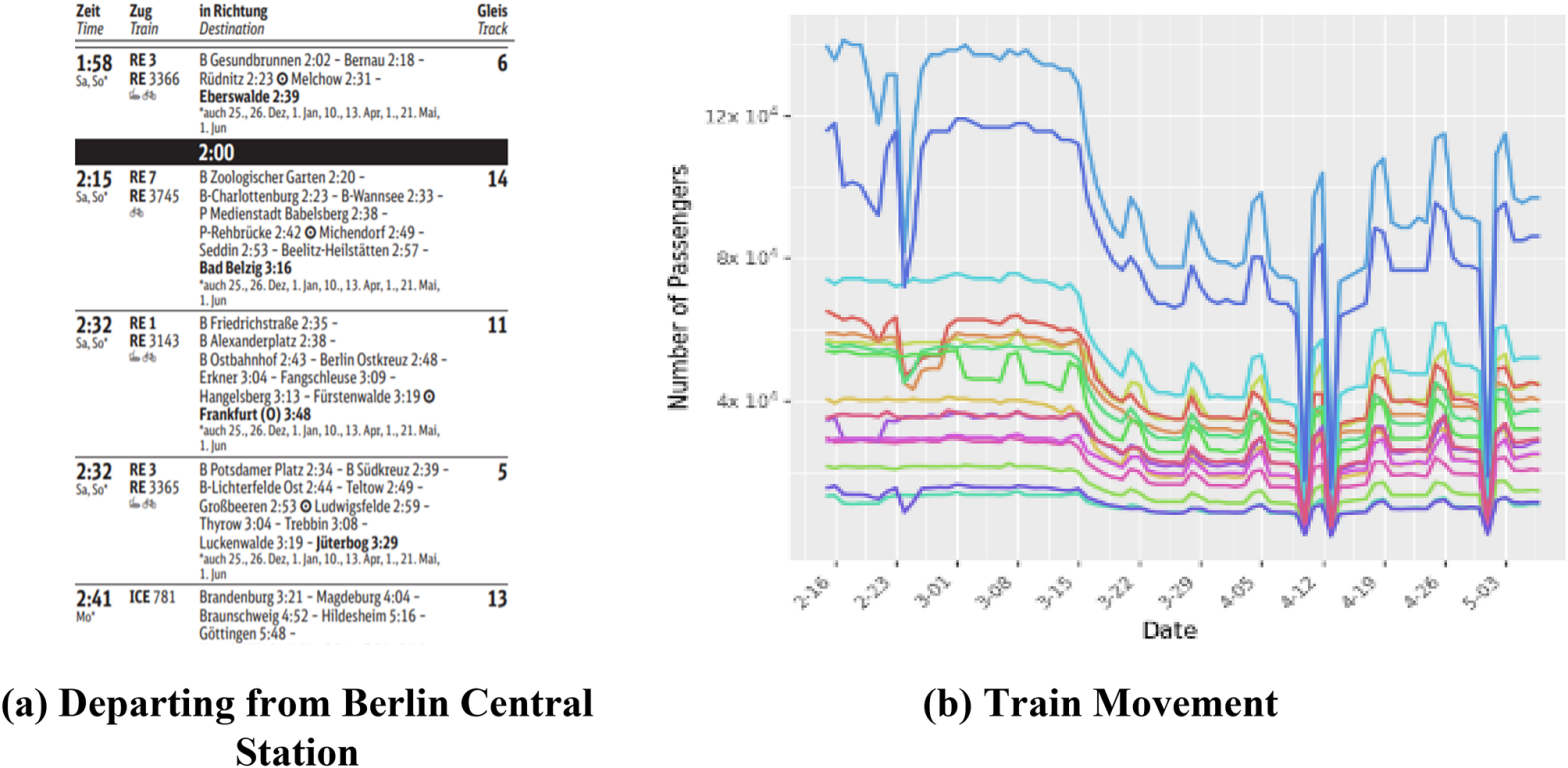
Ground transportation using trains in Germany. Panel (**a**) shows part of the train timetable available in all German train stations. More specifically, the sample snippet displayed in Panel (**a**) lists the departure times of trains leaving Berlin Hbf (Berlin Central Station). We parsed 538 timetable files to obtain the train schedule for all of Germany. We combine information from the arrival and departure timetables to construct the complete route of a train. Panel (**b**) shows the number of passengers arriving to different states by train.

We used Deutsche Bahn’s timetables (www.bahn.com) for all major train stations in Germany to estimate the number of daily rail travelers moving across states and arriving from neighboring countries. To account for the changes in movement due to COVID-19 and cancelations of several trains, we adjust the number of passengers moving across states by using the community mobility data for transit stations (Fig. 5).

We obtained the search history of a large European bus and train comparison platform to estimate the number of passengers moving across cities and states in Germany, and passengers traveling to Germany from neighboring countries. We set bus transport to zero after March 16, 2020 as all bus movement in Germany stopped on that day.

**Figure 6:**
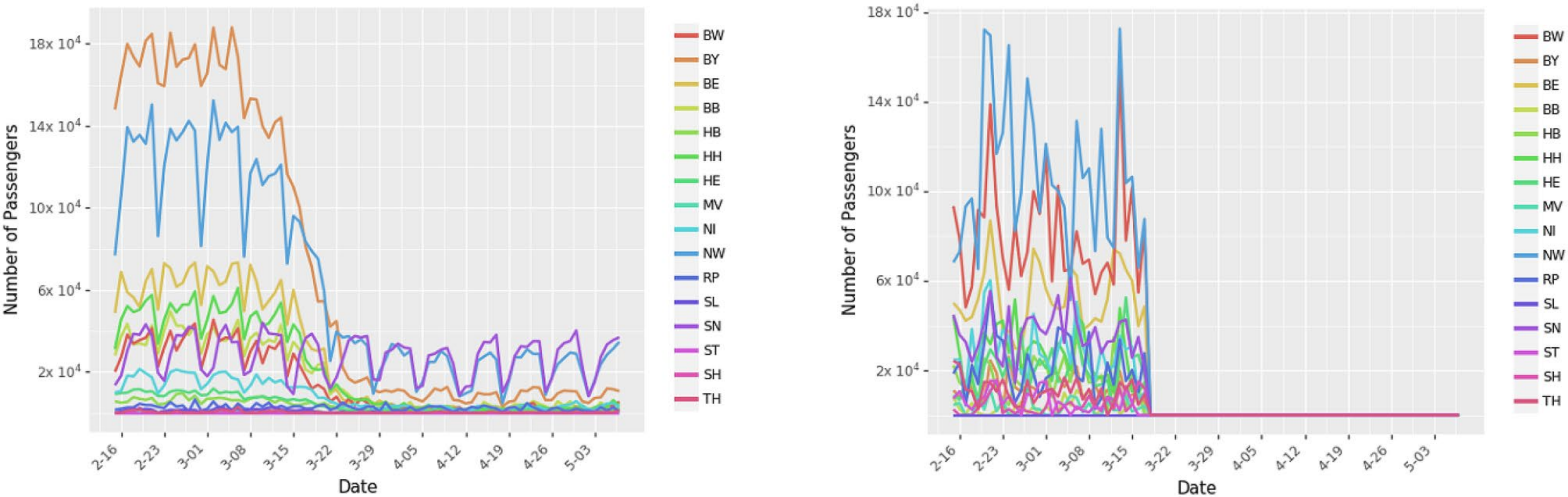
Transportation data in Germany. Panel (**a**) shows the number of air passengers arriving in different states in Germany. We assume 200 passengers for domestic and 500 passengers for international flights. Panel (**b**) shows the number of bus passengers arriving to different states from other states in Germany and neighboring countries. We assume 20 passengers per trip.

We also obtained flight transportation information from the Opensky Network^33^, whose database utilizes Automatic Dependent Surveillance Broadcast (ADS-B) flight trajectories to identify the departure and arrival airport of flights (Fig. 6).

**Figure 7:**
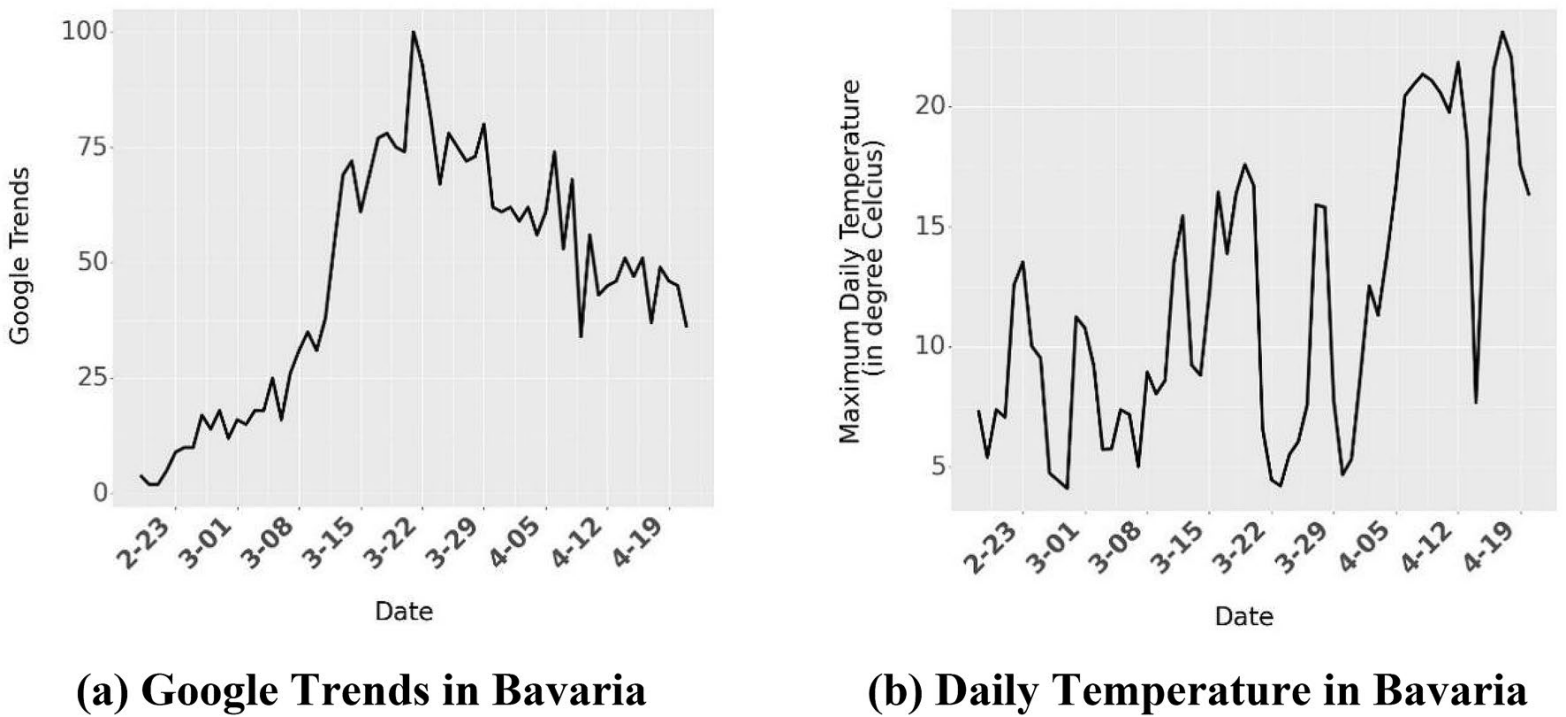
Covariates in Linear Regression Model. Panel (**a**) shows the Google Trends for the search term “COVID-19” for Bavaria. Google Trends numbers indicate search interest of a topic over time as a proportion of all other searches at the same time. We use Google Trends to account for increased awareness over time. Panel (**b**) shows the maximum temperature recorded in a day for Bavaria. We show the Google Trends and Maximum Daily Temperature for other states in the Supplementary Material (Figs. S8, S9 respectively).

We also use additional controls to isolate the effect of individual policies. We use Google Trends data for the search term “COVID-19” to control for awareness over time (Fig. 7a). Severe movement restrictions due to increased enforcement of these policy restrictions led to a greater sense of unease and dissatisfaction amongst some sections of the population (e.g.^34^, and while such protests are small, prolonged enforcement of restrictions could increase dissatisfaction). We use weather data (maximum daily temperature) from wetterkontor.de as a control to account for the propensity of the population to leave their homes as the weather improves (Fig. 7b). Finally, to account for unobserved heterogeneity across states and over time, we include state, week and day of week fixed effects. This allows us to control for variations that are not explicitly included in the model.

## Models

### Susceptible–exposed–infected–recovered (SEIR) model

The SEIR compartmental model is a transmission model of an infectious disease. The model includes a set of evolutionary equations that describes the spread of the disease in each subpopulation as follows:

1 $$\frac{d{S}_{i}}{dt}=-\frac{\left(1-\gamma *{sd}_{i}\right)\beta {S}_{i}{I}_{i}^{d}}{{N}_{i}}-\frac{\left(1-\gamma *{sd}_{i}\right)\mu \beta {S}_{i}{I}_{i}^{u}}{{N}_{i}}+{\sum }_{v}\left(\frac{{\theta }_{v}\sum_{j}{M}_{ij}^{v}{*S}_{j}}{{N}_{j}-{I}_{j}^{d}}-\frac{{\theta }_{v}\sum_{j}{M}_{ji}^{v}*{S}_{i}}{{N}_{i}-{I}_{i}^{d}}\right)$$

2 $$\frac{d{E}_{i}}{dt}=\frac{\left(1-\gamma *{sd}_{i}\right)\beta {S}_{i}{I}_{i}^{d}}{{N}_{i}}+\frac{\left(1-\gamma *{sd}_{i}\right)\mu \beta {S}_{i}{I}_{i}^{u}}{{N}_{i}}-\frac{{E}_{i}}{Z}+{\sum }_{v}\left(\frac{{\theta }_{v}\sum_{j}{M}_{ij}^{v}*{E}_{j}}{ {N}_{j}-{I}_{j}^{d}}-\frac{{\theta }_{v}\sum_{j}{M}_{ji}^{v}{*E}_{i}}{{N}_{i}-{I}_{i}^{d}}\right)$$

3 $$\frac{d{I}_{i}^{d}}{dt}=\frac{{\alpha }_{i}{E}_{i}}{Z}-\frac{{I}_{i}^{d}}{D}$$

4 $$\frac{d{I}_{i}^{u}}{dt}=\frac{(1-{\alpha }_{i}){E}_{i}}{Z}-\frac{{I}_{i}^{u}}{D}+{\sum }_{v}\left(\frac{{\theta }_{v}\sum_{j}{M}_{ij}^{v}*{I}_{j}^{u}}{{N}_{j}-{I}_{j}^{d}}-\frac{{\theta }_{v}\sum_{j}{M}_{ji}^{v}*{I}_{i}^{u}}{{N}_{i}-{I}_{i}^{d}}\right)$$

5 $${N}_{i}={N}_{i}+ {\sum }_{v}\left({\theta }_{v}{\sum }_{j}{M}_{ij}^{v}-{\theta }_{v}{\sum }_{j}{M}_{ji}^{v}\right)$$

where ${S}_{i}$, ${E}_{i}$, and ${N}_{i}$ represent the susceptible, exposed, and the total population of state $i$, respectively. ${I}_{i}^{d}$ represents the documented infected individuals which is the subset of the infected population that have symptoms severe enough to be diagnosed with the illness. ${I}_{i}^{u}$ is the rest of the infected population known as the undocumented infected individuals. We consider values for SEIR metapopulation state variables on day $t$. Additionally, the variable ${sd}_{i}$ (${sd}_{i}<1$) defines the daily change in social mobility (or degree of social distancing) in the state *i*. We also control for the potential of spillovers between states by accounting for interstate travel. Specifically, the number of interstate travelers from state $j$ to state $i$ via transportation network $v$, is ${M}_{ij}^{v}$ on a given day. We assume documented infected patients do not travel between states, while the asymptomatic undocumented infected individuals may move from one state to another. Finally, we note that the SEIR model works under the assumption that the incubation and infectious times are exponentially distributed and the relative change in the total population is negligible for the time period of the disease spread.

We estimate the parameters of this model via Ensemble Adjustment Kalman Filter (EAKF) which is suitable for models with a high number of parameters. We also adjust for various delays (e.g. the latency period from the onset of symptoms to diagnosis, the time between the exposure and becoming contagious, and the incubation period). Further details are provided in Supplement S1 and Table S1.

The parameters estimated from the SEIR model are used to predict the number of new case counts under the different scenarios we consider for policy (NPIs) easing.

### Linear regression: NPIs and social mobility

We use a linear regression model to estimate the association of NPIs with community mobility (${C}_{j,t}$) as shown in Eq. (6) where *K* is a constant. We use a binary variable ${x}_{j,p,t}=1$ if policy $p$ is active in state $j$ on day $t$, ${trend}_{j,t}$ is the exponentially smoothed Google Trends number for search term “COVID-19,” and ${temp}_{j,t}$ is the daily maximum temperature in degree Celsius in state $j$ on day $t$. To account for state-level unobserved heterogeneity, we use state fixed effects ($stat{e}_{j}$). In addition to state-level differences, we also control for week-based differences and day of week-based differences by incorporating week fixed effects ($wee{k}_{t}$) and day of week fixed effects ($da{y}_{t}$). For stable parameter estimation, we consider state Thuringia as our base state (0 state fixed effects), week 9 as our base week (0 week fixed effect) and Monday as our base day (0 day of week fixed effect). Fixed effects in Eq. (6) are modeled as indicator functions. (details in Supplement Section S3).

6 $${C}_{j,t}=K+{\sum }_{p=1}^{7}{{\beta }_{p}^{policy}x}_{j,p,t}+{\beta }_{t}^{trend}{trend}_{j,t}+{\beta }_{m}^{temp}{temp}_{j,t}+{\sum }_{j=1}^{16-1}{\beta }_{j}^{state}stat{e}_{j}+{\sum }_{w=1}^{9-1}{\beta }_{w}^{week}wee{k}_{t}+{\sum }_{d=1}^{7-1}{\beta }_{d}^{day}da{y}_{t}+{\epsilon }_{j,t}$$

This specification models states to be independent, which may lead to failure of the SEIR model in capturing spillovers across states. To address spillovers from different states, we use movement across states in the SEIR model (car, bus, train, truck, and flight) and state fixed effects. We assume that Google Trends might be able to capture spillover effects (if cases increase in one state, population in neighboring states will be more self-aware). Further details are presented in Supplement S3.3.

### Limitations: association as opposed to identification

Our approach rests on spatial–temporal variations in NPIs contributing to variations in social distancing, which in turn is linked to variations in COVID-19 case numbers. We *do not claim causality* when analyzing this chain of events, as there are ways in which causal identification may fail. For example, if the data do not provide adequate variation in the sequencing of policy interventions, then our estimates cannot uniquely identify the effect of each intervention. Figure 3 shows for example that Contact Restriction never occurred first in any state, and thus our associative effect estimate likely contains bias. Combinatorically, for the seven NPIs observed, there exist 13,700 possible sequences (i.e., there are ${\sum }_{k=0}^{7}7Pk=\mathrm{13,700}$ possible orders/permutations in which the seven NPIs are implemented in each state). However, in real world settings, most countries adopted similar sets of policies within a short span of time despite these vast sets of possibilities. For example, Sebhatu et al.^5^ note that “almost 80% of OECD countries adopted the same COVID-19 NPIs within a span of 2 w[ee]k[s].” They find that rather than accounting for country specific characteristics, policymakers set policies based on “…the number of earlier adopters in the same region.” Due to the homogeneity of policies despite the heterogeneity of country and state characteristics, all policy level analyses suffer from estimation biases due to the lack of variation in the adoption and implementation of policies. Even though we observe temporal and sequencing differences across states, the variation is not sufficient to attribute causality to these interventions. Ultimately, we would like to examine interventions at a more granular, perhaps even individual level (e.g., assess the probability of a person contracting COVID-19 when certain NPIs are enacted) to obtain a greater variation in the sequencing of interventions. Unfortunately, this is not how policy decisions are made: they cover wider jurisdictions, and their historical sequence is limited.

A second thread to identification are spillover effects across states that may result in biased estimates. We try our best to account for spillovers between states in the following ways: (1) implementing most interstate movement in the SEIR model (car, flight, bus, train, and truck), (2) include Google Trends in the linear regression model (people in a state would be more cautious and search for “COVID-19” more frequently if case numbers rise in neighboring states), and (3) include state fixed effects to account for state level heterogeneity (to help alleviate potential spillover of a particular state from its neighbors). Moreover, Supplement S3.3 addresses the issue of potential spillover across states in more detail by evaluating two alternative specifications. However, we still cannot completely rule out the possibility of spillovers.

Due to the data-imposed inability to identify the causal impact of NPIs on COVID-19 cases, this study only proposes associative effects for each NPI.

## Results

### Quantifying policy contributions

**Figure 8:**
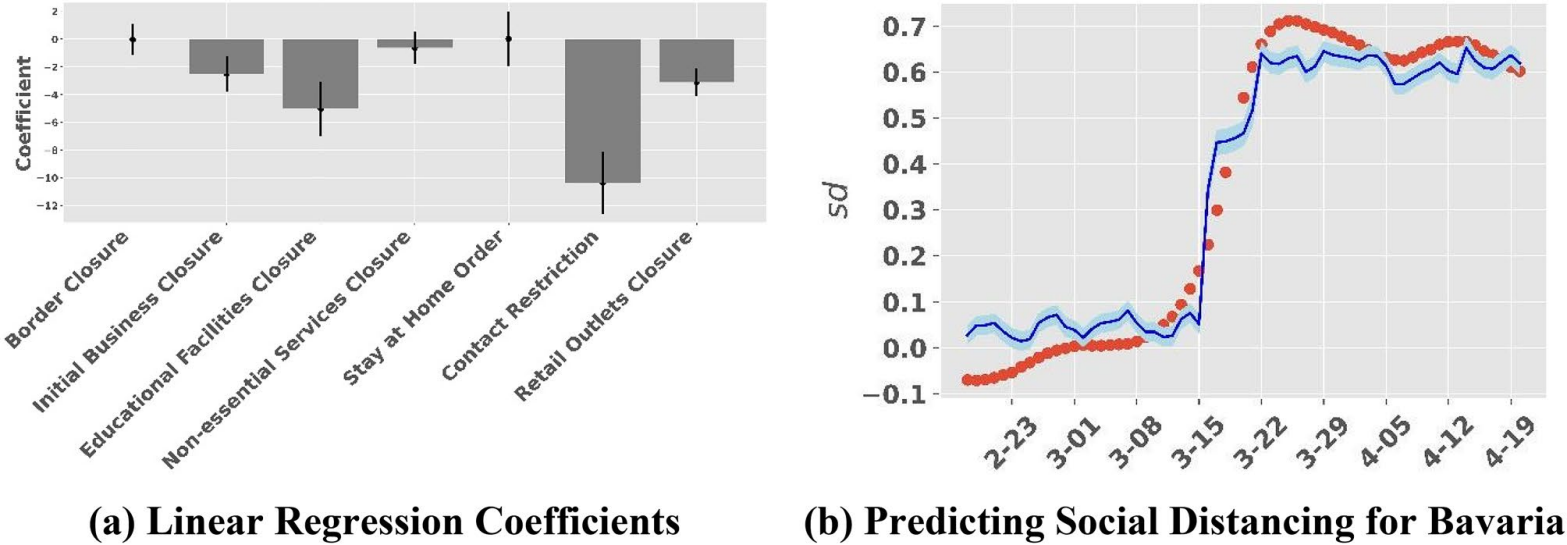
Linear regression model for estimating the marginal contribution of different NPIs on social distancing. Panel (**a**) shows the linear regression coefficients for different NPIs. The vertical bars show the 95% confidence interval bounds for coefficients. Our regression model has an R-square value of 0.976. Panel (**b**) shows the smoothed social distancing (red dots) and predicted social distancing (blue line) in Bavaria. The blue shade shows the 95% confidence bounds around prediction for social distancing. The plots for all other states are included in the Supplementary Material (Fig. S11).

To determine the impact of different state policies, we use a linear regression model to predict changes in mobility (*C*_*j,t*_) due to policy *p*. Coefficients from the linear regression model are shown in Fig. 8a. We calculate social distancing (*sd*_*j,t*_) from mobility in Fig. 8b. As explained above, we *cannot identify* each policy’s *causal effect* on reducing disease spread. However, we are still able to provide meaningful insights of each policy’s contribution in reducing the disease spread.

Figure 8a shows the parameter estimates of various NPIs on social mobility. The complete list of parameter estimates are provided in the Supplement (Table S3). The estimates capture the average incremental effect of these policies across Germany’s 16 states in reducing mobility. From the graph, we see that contact restrictions have a very large negative effect on mobility (10.3 percentage point drop in mobility, 95% Confidence Interval (CI): 8.2 to 12.5). COVID-19 is mainly spread through airborne transmission among individuals in close proximity. The policy of contact restrictions limits the number of people that may congregate and requires all people to stay a minimum of 6 feet apart from one another to ensure social. Educational Facilities Closure also precipitates a significant drop in mobility (5 percentage point drop; CI: 3.1–6.9). A significant drop is possible as it indirectly leads to decrease in mobility (for example, children staying home without care givers forces parents to work from home). The other policies listed in order of impact on mobility (estimate of percentage point drop in mobility from the regression and 95% Confidence Interval in parenthesis) are: Retail Outlets Closures (3.1, CI: 2.1–4.1), Initial Business Closures (3.4; CI: 1.2–3.7), Border Closures (0.03, CI: − 1 to 1), Non-essential Services Closures (0.62; CI: − 0.5 to 1.7), and Stay at Home Orders (− 0.01; CI: − 1.9 to 1.8). It can be observed that parameter estimates for Border Closures, Non-essential Services Closure, and Stay at Home Orders are statistically insignificant (confidence intervals include 0). However, it does not conclude that these NPIs had no effect. For example, international travel might have played a significant role in seeding the disease. However, once the Borders were closed, the Border Closure NPI played a minimal role in mitigating the spread of the disease. As discussed in the previous section (“Limitations: Association as opposed to Identification”), we estimate associations as opposed to causal effects.

### Predicting disease spread

We use the *predicted* mobility from the linear regression to predict new case counts. We investigate the contribution of each policy to the mitigation of disease spread by determining the role of social distancing in the estimation of the number of susceptible and exposed individuals in a given population. We modify the SEIR model used in^20^ to include different transportation networks and predicted mobility for each state (Eqs. 1–5). Using the estimation procedure in^20^, we find the model parameters to predict disease spread for all 16 states in Germany—the model accounts for documented as well as undocumented infected cases. As shown in Supplementary Material Fig. S20, the proportion of documented infected (${I}^{d}$) as a function of total cases increases over time. This finding aligns with expectations because of the rapid increase in testing across Germany^35^.

**Figure 9:**
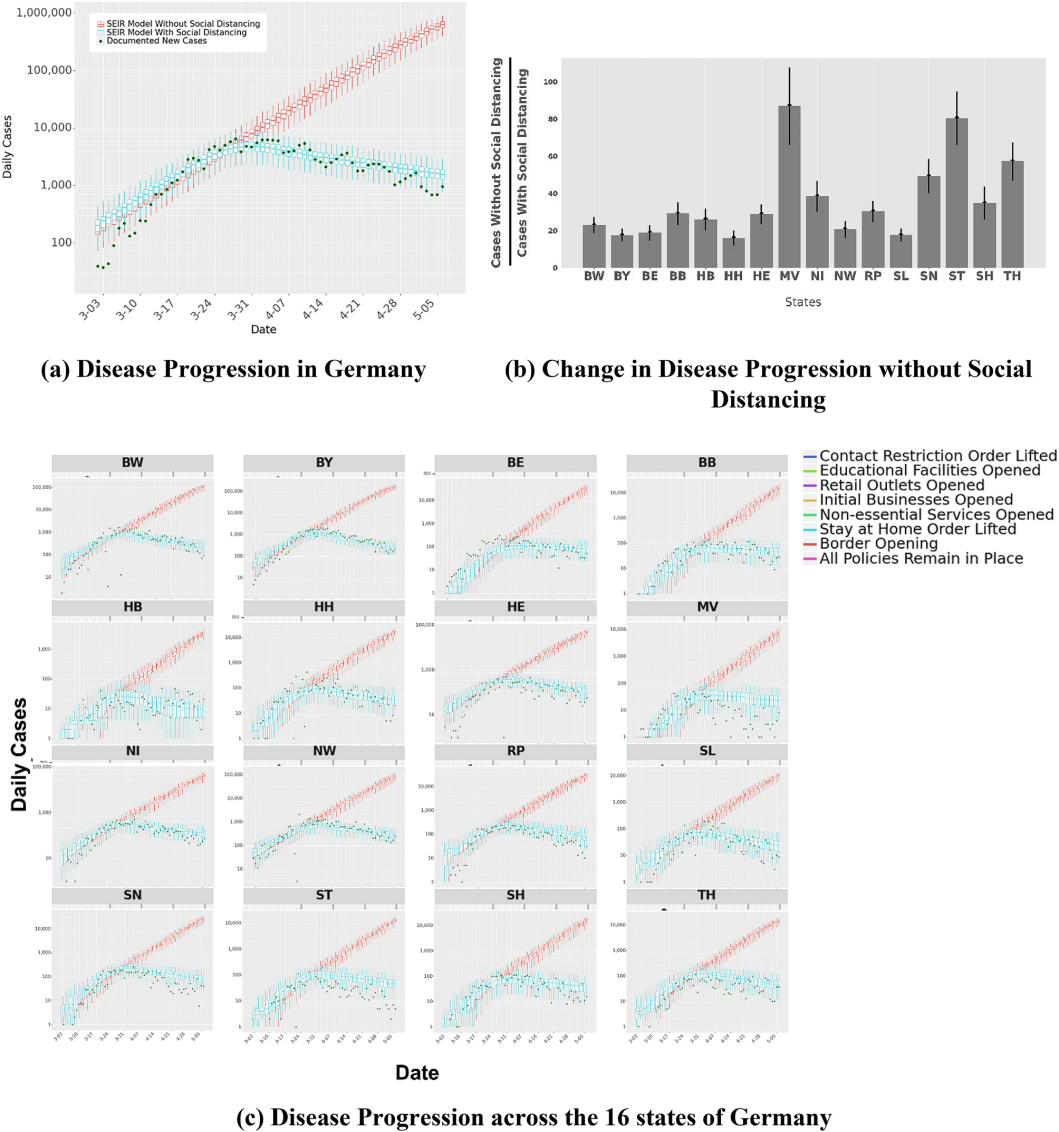
SEIR Model Predictions. Panel (**a**) shows the predictions of new case counts in Germany with and without any distancing measures, against actual cases for the same time period. Panel (**b**) presents the expected increase of cumulative cases (in multiples) when there is no social distancing as compared to the actual cases numbers. Panel (**c**) plots the predictions of new case counts with and without any distancing measures, against actual case numbers for all states. The box and whiskers provide the median, interquartile range, and up to a maximum of 1.5*IQR from the box hinges derived from 4000 simulations using the inferred model parameters. Our predictive accuracy is higher for states with high case numbers such as Baden-Württemberg (BW) and Bavaria (BY) than for the small city-states Berlin (BE), Bremen (HB), and Hamburg (HB). We note that the last 17 days of Panels (**a**,**c**) correspond to out-of-sample forecasts from the SEIR model.

Figure 9a shows the actual disease progression in Germany; the disease spread as predicted by our model in the presence of predicted social distancing, as well as disease spread as predicted by our model when mobility remained unchanged with no social distancing measures. Similar predictions for the states are provided in Fig. 9c. We use the time period of Feb 18, 2020–Apr 20, 2020 to infer model parameters. This period includes early stages of the *COVID-19* epidemic in Germany and the time that state policies are enacted. We use these parameters to estimate the number of daily documented cases during the time interval of Feb 18, 2020–May 7, 2020, which corresponds to 17 days out of sample forecasts. The model finds 172,922 (IQR: 140,301–204,952) cumulative documented cases in Germany as of May 7, 2020 (actual reported cases: 166,069) with the estimated average error rate of 4%. Figure 9b shows the amount of expected increase in the number of cases across the states of Germany and the nation, if no social distancing was practiced. Across Germany one would expect a 24.6-fold (IQR: 20–29) increase in the number of cases without any social distancing (i.e., $s{d}_{i}=0$), the effect varying significantly by states from a low of 16-fold (IQR:12.2–19.7) in Hamburg to a high of 86-fold (IQR: 66.3–107.3) in Mecklenburg-Vorpommern.

### Lifting of restrictions

We simulate what-if scenarios to determine the impact of lifting restrictions on new cases in each state. Under the scenario that a restriction has been relaxed while others remained operational, we forecast mobility using Eq. (6), and we subsequently project the new case count using the predicted mobility. This step is repeated across all restrictions, each relaxed individually.

**Figure 10:**
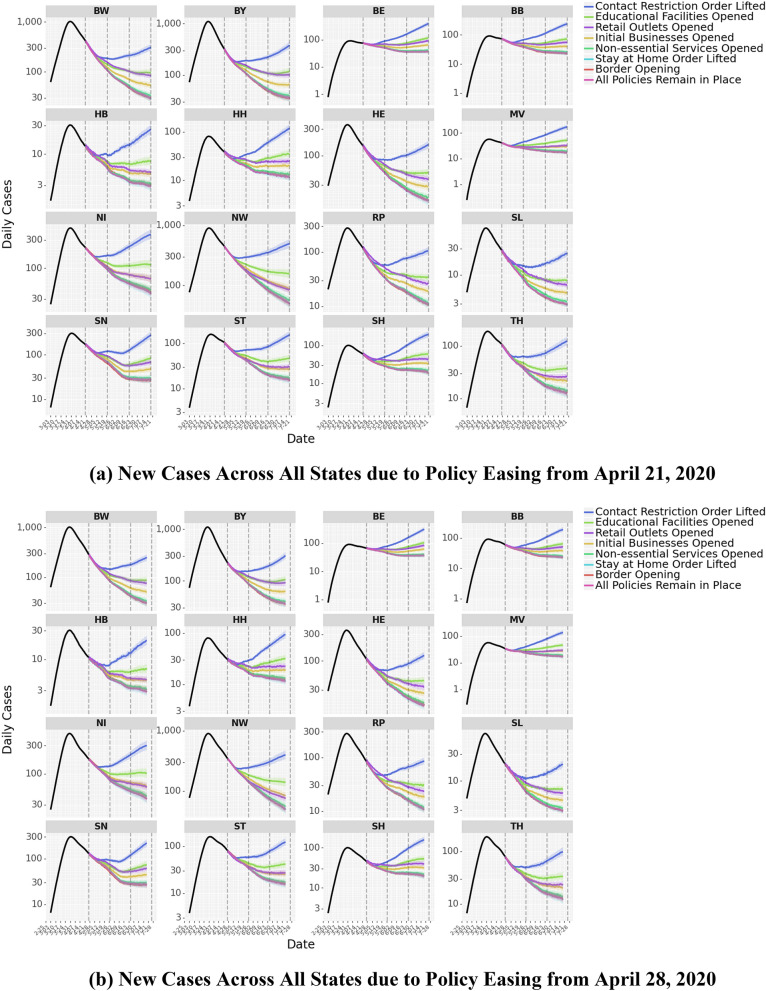

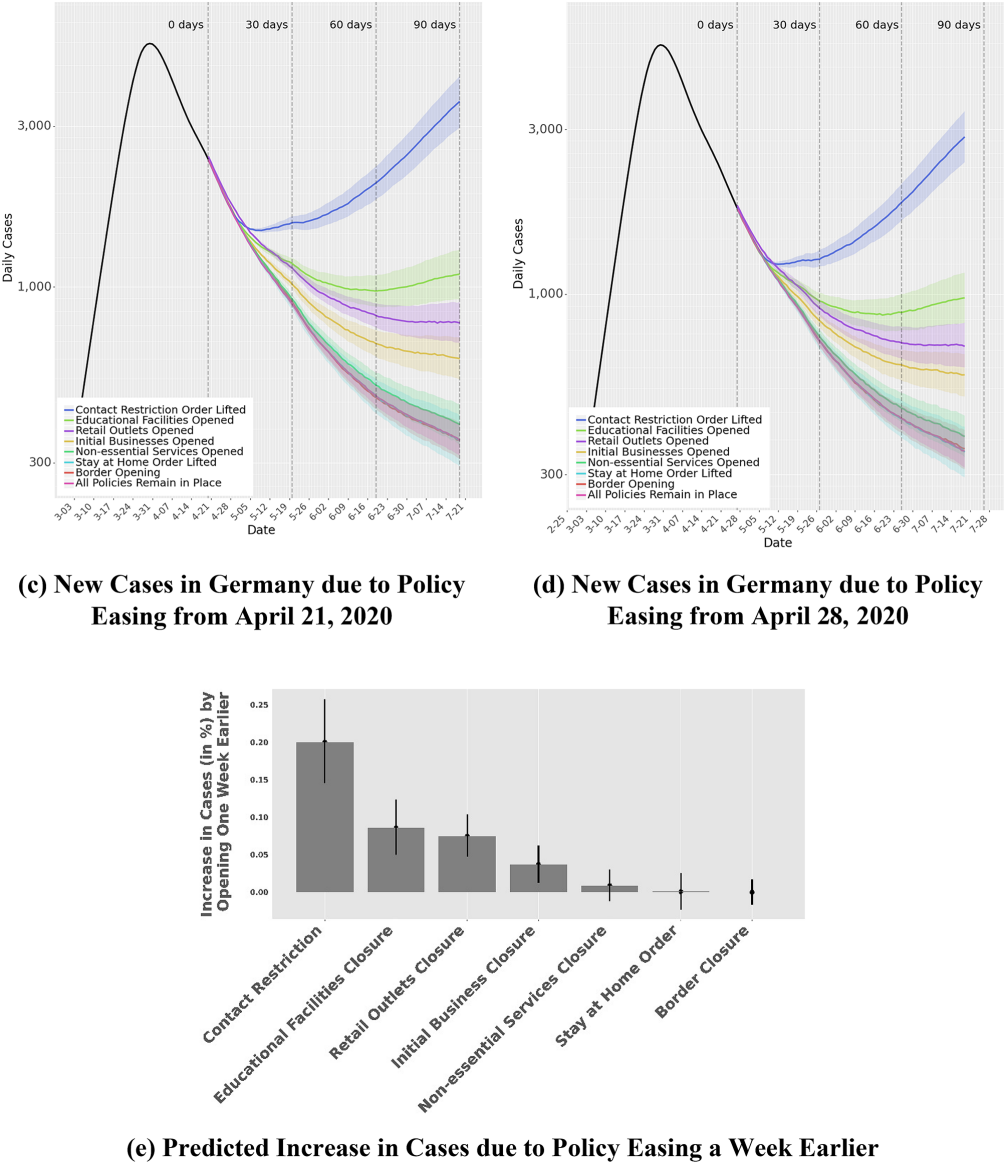
Lifting of Policy Restrictions. Panels (**a**,**b**) show the predictions of new case counts across all states of Germany due to lifting of policy restrictions from April 21, 2020 to July 19, 2020. In Panel (**a**) policy restrictions are relaxed, one-at-a-time on April 21, 2020, in Panel (**b**) policy restrictions are relaxed, one-at-a-time on April 28, 2020. Panels (**c**,**d**) illustrate the same for all of Germany. Panel (**e**) charts the predicted increase in cases due to policy easing a week earlier.

In *Scenario 1*, we project all changes from April 21, 2020 to July 19, 2020, assuming that the restriction was relaxed on *April 21, 2020*. Figure 10a,c show the projections of case counts over a 90-day period if a restriction was relaxed exclusively. Figure 10b,d show *Scenario 2*, which projects all changes from April 21, 2020 to July 19, 2020, assuming that the restriction was relaxed on *April 28, 2020*. Because the two scenarios are exactly one week apart, it allows us to determine the impact of delaying the lifting of a restriction by 1 week. From the analysis, the lifting of contact restrictions, i.e., the rule limiting movement in public spaces, had the biggest impact on new case counts. Compared to keeping the restrictions in place, lifting contact restrictions is associated with a 150% (IQR: 144–156%) increase in daily case numbers in Scenario 1 and a 108% (IQR: 103.7–112.5%) increase in Scenario 2. However, lifting educational facilities closure is associated with an increase in daily case numbers by 46.1% (IQR: 44.0–48.1%) in Scenario 1 and 34.4% (IQR: 32.7–36.2%) in Scenario 2. Opening of retail outlets is linked to a 33.9% (IQR: 33.0–34.8%) increase in daily case numbers in Scenario 1 and a 24.5% (IQR: 23.4–25.6%) increase in Scenario 2. Lifting restrictions on initial business closures is associated with an increase in daily case numbers by 18.6% (IQR: 17.8–19.5%) in Scenario 1 and 14.4% (IQR: 13.7–15.0%) in Scenario 2, and easing non-essential service closures is associated with an increase in daily case numbers by 3.6% (IQR: 3.1–4.1%) in Scenario 1 and 2.6% (IQR: 2.2–3.0%) in Scenario 2. These results show that NPIs have differential impacts on lowering disease spread and suggest a measured approach to lifting restrictions. For example, the opening of retail outlets could be balanced by maintaining the restrictions around limiting the number of individuals in a given place or store (e.g., controlling entry)—thereby allowing for the resumption of economic activity while limiting the risk of contagion.

Figure 10e shows the increases in the expected number of cases if a restriction was lifted on April 21 versus on April 28. Delaying the lifting of certain restrictions by one week could also have a significant impact on the total case counts. This occurs not only due to the delay, but also because the number of infected individuals that a person could come in contact with decreases over the week. For example, delaying the lifting of contact restrictions by one week is associated with a reduction in the number of new cases over the 90-day forecast by an average of 20% (IQR: 14.6–25.7%). We also observe that lifting restrictions on educational facilities closure and retail outlets closure is linked to an average 8.9% (IQR: 5.1–12.2%) and 7.5% (IQR: 4.8–10.2%) increase in total case numbers over the 90-day forecast period.

## Discussion

This study explores the role of NPIs in reducing the spread of COVID-19. We extend the spatio-temporal SEIR model in^20^ by incorporating daily social distancing numbers from transportation data and mobility patterns. While our analysis does not allow for causal identification, each policy’s association with the mitigation in disease spread provides meaningful insights. We do this by first relating the NPIs to social mobility changes across the 16 states. Next, we link these changes in social mobility to the spread of COVID-19 by reconstructing patterns of disease spread across Germany’s 16 states. Using this link with social mobility changes, we investigate the marginal association of each of the various NPIs implemented by state governments in Germany with disease mitigation. The model suggests that without NPIs, COVID-19 cases may have shown a 24.6-fold increase across Germany as of May 7, 2020. Finally, we forecast the number of new cases when policies are relaxed one at a time, suggesting that certain policies have a larger impact on disease spread than others. Our model forecasts suggest that early relaxation of some NPIs could result in an increase in the number of cases, potentially leading to a future wave. This observation is confirmed by an estimated increase in the effective reproduction number ${R}_{e}$ (in the Supplementary Material). We also compare case counts for policies relaxed with a one-week delay; keeping some NPIs in place for an extra week is associated with a reduction in COVID-19 cases by up to 20% (as of July 19, 2020). The results confirm that policy restrictions are not all equal in their ability to affect disease spread. The policy of restricting mass gatherings (Contact Restriction) is estimated to be the most effective NPI to contain COVID-19, followed by closures of various businesses and stay at home orders. Due to this variation in effect, it is advisable to lift restrictions with minimal effects first, gradually easing restrictions that potentially lead to higher case numbers. This study presents a comprehensive quantitative analysis that includes individual effects of NPIs on the transmission of COVID-19. To the best of our knowledge this is the first study that uses variations in policy interventions by governments to discover their differential impacts at reducing mobility, which in turn reduces disease spread. Prolonged lockdowns and restrictive policies can have devastating social and economic consequences; however, opening too soon could result in rapid disease spread. Therefore, governments need to develop cautious approaches to lifting restrictions to return to normalcy^36^. The approach presented in this paper allows for a deeper understanding of the policy effects on mitigating the spread of COVID-19. The forecasts of disease spread when NPIs are partially loosened guide policymakers towards the appropriate strategy when reversing restrictions.

## Supplementary information

Supplementary Information.

## Data Availability

All code and data are available in the supplementary materials and posted online in a public repository https://github.com/ehgh/COVID-19-case-estimation-and-policy-effects.
