# Supplementary material for: Preparing for a future COVID-19 wave: insights and limitations from a data-driven evaluation of non-pharmaceutical interventions in Germany
Source: Sci Rep. 2020 Nov 18;10:20084. doi: 10.1038/s41598-020-76244-6 (PMC7674458; doi:10.1038/s41598-020-76244-6)
Supplement: Supplementary file 1 — Supplementary Information. [file 41598_2020_76244_MOESM1_ESM.docx]

**Preparing for a Second COVID-19 Wave:**

**Insights and limitations from a data-driven evaluation of non-pharmaceutical interventions in Germany**

**Supplementary Material**

Ashwin Aravindakshan, Jörn Boehnke, Ehsan Gholami and Ashutosh Nayak

University of California, Davis

**1. Model Configuration and Initialization**

 Following [1], the SEIR transmission model integrates the effect of human mobility on disease spread through a set of evolutionary equations. The model is given below:

$\frac{dS_{i}}{dt}=-\frac{\left( 1-\gamma*{sd}_{i} \right)\beta S_{i}I_{i}^{d}}{N_{i}}-\frac{\left( 1-\gamma*{sd}_{i} \right)\mu\beta S_{i}I_{i}^{u}}{N_{i}}+\sum_{v} \left( \frac{\theta_{v}\sum_{j} M_{ij}^{v}{*S}_{j}}{N_{j}-I_{j}^{d}}-\frac{\theta_{v}\sum_{j} M_{ji}^{v}*S_{i}}{N_{i}-I_{i}^{d}} \right)$ (S1)

$\frac{dE_{i}}{dt}=\frac{\left( 1-\gamma*{sd}_{i} \right)\beta S_{i}I_{i}^{d}}{N_{i}}+\frac{\left( 1-\gamma*{sd}_{i} \right)\mu\beta S_{i}I_{i}^{u}}{N_{i}}-\frac{E_{i}}{Z}+\sum_{v} \left( \frac{\theta_{v}\sum_{j} M_{ij}^{v}*E_{j}}{N_{j}-I_{j}^{d}}-\frac{\theta_{v}\sum_{j} M_{ji}^{v}{*E}_{i}}{N_{i}-I_{i}^{d}} \right)$ (S2)

$\frac{dI_{i}^{d}}{dt}=\frac{\alpha_{i}E_{i}}{Z}-\frac{I_{i}^{d}}{D}$ (S3)

$\frac{dI_{i}^{u}}{dt}=\frac{(1-\alpha_{i})E_{i}}{Z}-\frac{I_{i}^{u}}{D}+\sum_{v} \left( \frac{\theta_{v}\sum_{j} M_{ij}^{v}*I_{j}^{u}}{N_{j}-I_{j}^{d}}-\frac{\theta_{v}\sum_{j} M_{ji}^{v}*I_{i}^{u}}{N_{i}-I_{i}^{d}} \right)$ (S4)

$N_{i}=N_{i}+ \sum_{v} \left( \theta_{v}\sum_{j} M_{ij}^{v}-\theta_{v}\sum_{j} M_{ji}^{v} \right)$ (S5)

where $S_{i}$, $E_{i}$, and $N_{i}$ represent the susceptible, exposed, and the total population of state $i$, respectively. $I_{i}^{d}$ represents the documented infected individuals which is the subset of the infected population that have symptoms severe enough to be diagnosed with the illness. $I_{i}^{u}$ is the rest of the infected population known as the undocumented infected individuals. We consider values for SEIR metapopulation state variables on day $t$. For the rest of this paper, we omit the time index $t$ that represents the time dependency of variables. $\beta$ is the transmission rate of the disease from a documented infected individual to a susceptible individual under normal population mobility. The transmission rate due to undocumented infected is captured by $\mu\beta$ with $\mu$ being the reduction coefficient ($\mu<1)$. Additionally, the variable ${sd}_{i}$ (${sd}_{i}\in[-1, 1]$) defines the daily change in social mobility (or degree of social distancing) in the state *i*, and the coefficient $\left( 1-\gamma{*sd}_{i} \right)$ is the decrease/increase in transmission rate due to changes in socializing and population mobility ($\gamma\leq1$). The ratio of documented to total infected individuals in the state $i$ is $\alpha_{i}$, which varies among states with dissimilar population demographics based on age and gender [2]. $Z$ is the average incubation time, and $D$ is the infection period. More precisely, $D$ captures the effective period in which the infected individual moves out of the chain of disease transmission by perishing, recovering, or entering quarantine. The number of interstate travelers from state $j$ to state $i$ via transportation network $v$, is $M_{ij}^{v}$ on a given day, with $\theta_{v}$ to fix the underreporting of transportation ${(\theta}_{v}\geq1)$. Two transportation networks represented by $G$ and $A$ account for ground and air mobility. The ground transportation $M_{ij,G}$ includes movement of individuals by four different sub-networks: $M_{ij}^{G}=\sum_{g} M_{ij}^{g}$. These sub-networks are: cars, trains, trucks and buses. Similarly, $M_{ij}^{A}$ is the number of people traveling via flights. In this model, we assume documented infected patients do not travel between states, while the asymptomatic undocumented infected individuals have the ability to move from one state to another.

We estimate the parameters of this model using the procedure described in [1]. The model parameters of the SEIR model are inferred via iterative filtering of stochastic ensemble Kalman filter (EnKF-IF). The EnKF is a Monte Carlo (MC) approximation of the Kalman filter. We specifically used the Ensemble Adjustment Kalman filter (EAKF) which is suitable for models with a high number of parameters. In this technique, in each iteration, a presumably Gaussian distributed ensemble of state vectors is adjusted to posterior distribution via Bayes rule. The state vector includes model parameters and meta-population values. We use the maximum likelihood approach to determine the final values of the state vector in the algorithm. The daily documented cases act as observations in the model. We find that a few hundreds of ensembles are sufficient to accurately infer the model parameters.

We introduce a randomly drawn number for detecting delay of each documented case. This additional delay captures the latency period from the onset of symptoms to diagnosis and the time it takes an individual to become contagious from an initial exposure (2). The model randomly adds delay with Gamma-distributed values of shape $a=1.78$ and average of $T=6$ days. This distribution fits with the information from confirmed cases in China and South Korea [1,5,6]. We examined Gamma distributions with various averages and a constant shape to capture the most accurate average delay time for estimation purposes.

Furthermore, we introduce another delay for the effect of social distancing on daily confirmed cases. There is an average of 6 days delay between the change in human mobility (${sd}_{i}$) and the corresponding change in the number of daily documented infected cases across all states. This constant shift accounts for the time that the change in mobility starts showing an effect on disease transmission rate.

The configuration of model and initial ranges for parameters and values are represented in Table S1. The EAKF algorithm is not limited by parameter prior range and can move outside of prior range to find the optimum solution. We choose a suitable prior range for state vector initial values to facilitate the convergence while covering most of the possible values for parameters.

The prior range of $\mu$ covers a wide range of possible values [0, 1]~~.~~ The prior range for $\alpha$ is set to include most of its possible values (0,1] with lower bound set to 0.5 to account for the high volume of Covid-19 testing in Germany. The prior range for $\beta$ is set to cover a wide range of values for $R_{0}$, i.e. [0, 12]. Prior range of $Z$ and $D$ are chosen to cover the known average incubation and infection period for Covid-19 [3, 5]. Prior range of $\theta$ is set to capture most of the possible range of underreporting of transportation. The range limits the number of each state’s travelers to its population (Figure S5, S6, S7 in the manuscript). The prior range for $\gamma$ covers most of the possible range for the effect of human mobility and social distancing on transmission rate, i.e. (0, 1]. The lower bound allows for at least a 50% drop in transmission rate if social mobility is dropped 100%.

We use Feb 18, 2020 – Apr 20, 2020 as the time period for model inference. Feb 18, 2020 is the early stages of *COVID-19* epidemic in Germany with only two states with reported cases. The initial susceptible population$S_{i}$ of each state is set to its initial total population. The initial documented cases, $I_{i}^{d}$, is set to reported cases on Feb 18, 2020. The exposed and undocumented infected initial values are set in the following way. Based on reported daily cases, there are three states that are early hubs of Covid-19 in Germany. Nordrhein-Westfalen (NW) is the pioneer state linked to large carnival events, followed by states Baden-Wurttemberg (BW) and Bayern (BY) via outbreak in Italy (2). The first large cluster of reported cases in Nordrhein-Westfalen was reported on Feb 28, 2020 with 25 cases. Accounting for the average doubling time of 6.4 days [95% CI: 5.8-7.1 days] for cases (4) and an average of 86% [95%CI: 83%, 90%] undocumented cases in early stages [1], we estimate to have [95% CI: 390-825] cases on the initial day of the model in Nordrhein-Westfalen. We set the initial value range for $E_{NW}$, $I_{NW}^{u}$ to be [0-400] and uniformly select random values from this range. Similarly, we set the initial range of $E_{BW}$, $I_{BW}^{u}$, and $E_{BY}$, $I_{BY}^{u}$for Baden-Wurttemberg and Bayern to [0, 200]. Excluding the aforementioned states, the rest of the German states’ initial values for $E$and $I^{u}$were drawn uniformly from [0, $C_{max}$] with $C_{max}$ being the aggregated number of undocumented infected travelers from the three hub states Nordrhein-Westfalen, Baden-Wurttemberg, and Bayern on the day of Feb 18:

${C_{i}}_{max}^{t=0}=\sum_{v} \sum_{i\in\{BW,BY,NW\}} M_{ji}^{v}E_{i}/N_{i}$ (S6)

All ensembles’ initial state vector values are drawn randomly via Latin hypercube sampling with uniform distribution from the initial ranges.

The histograms of inferred model parameters are shown in Figure S1. The model parameter values selected from the set of inferred parameters are depicted by red lines in Figure S1. Please note that parameters sets are complex and while one parameter might have a high inferred frequency in a set, another parameter in that set might not have the highest inferred count.

**2. Data Collection**

**2.1 Observations of confirmed COVID-19 Cases**

The daily number of confirmed COVID-19 cases in Germany have been collated from data released by the Robert Koch Institute (RKI) in Berlin, Germany. Figure S2 shows how COVID-19 spread across Germany over time. When individuals contract COVID-19, they may remain asymptomatic. During this period, they are active carriers of the virus. Symptomatic and asymptomatic individuals are identified through clinical testing. In Germany, a primary care physician’s referral is required for COVID-19. More than 100 laboratories were contracted to conduct testing for the COVID-19 virus. The daily cases in RKI includes the number of confirmed positive cases.

The RKI published the daily case reports at 10:00 prior to March 1, 2020. Between March 1, 2020 and March 9, 2020, the RKI reported cases at 10:00 and at 15:00. Starting March 10, 2020, due to continuously rising case numbers, RKI switched to adopting the numbers that were electronically transmitted from testing centers across Germany. The new case numbers were published once at 15:00. Starting March 17, 2020, the daily cases are published at midnight for the previous day. To account for the discrepancy in reporting time, we used a constrained cubic spline interpolation method to obtain the number of cases prior to March 17, adjusted for midnight reporting. Daily cases using cubic spline interpolation is shown in Figure S3.

**2.2. Community Mobility Data Trends**

Google aggregates data from users’ anonymized location history (for users who switched on the location history settings in their android mobile phones) to estimate foot traffic across six different location categories -- retail and recreation, groceries and pharmacies, parks, transit stations, workplaces, and residential (Figure S4). With the outbreak of COVID-19, Google released data for changes in foot traffic (in percent points) for the six location categories from February 15, 2020. These percentage changes in foot traffic are reported as community mobility trends in the reports. We use trends in retail and recreation to measure social distancing.

**2.3 Mobility Data**

COVID-19 started in Wuhan, China, and spread to Germany and other parts of the world through cross-border human movement. We use air and different types of ground transportation to accurately collect the movement data across different states in Germany. For international travel into Germany from other countries we consider traffic from 142 countries (including nine countries that share international borders with Germany) through ground and air transportation. Next, we discuss the movement data for different models.

**2.4 Car Mobility Data and Truck Mobility Data**

We collected detailed five-year highway traffic data from Jan 1, 2013 to Dec 31, 2018, provided by the German Bundesanstalt für Straßenwesen (Federal Institute for Roadways). The dataset contains the hourly count of vehicles passing through one of about 2,800 automatic counting stations along highways and state streets. Each station records the flow of traffic in the two directions ($z_{1}$ and $z_{2})$. The dataset contains geographical coordinates of the locations for these checkpoints. Sensors were used to identify the vehicles as cars, buses and trucks. We use correction factors for public holidays, day of the week and state population to extrapolate hourly traffic for Jan 1, 2020 to May 7, 2020. We add the hourly data to get the daily movement data. We calculate the mean of daily movement data ($W_{h},z_{1},z_{2}$) for highway $h$(for both directions). We also calculate the mean of the movement data for each highway during public holidays ($W_{h}^{ph},z_{1},z_{2}$) and day of the week ($W_{h}^{dw},z_{1},z_{2}$). We use the ratio, ($W_{h}^{ph},z_{1},z_{2}$)/ ($W_{h},z_{1},z_{2}$) as correction factor for public holidays. We also use similar correction factor for day of the week. Finally, we correct for increase in population by using the ratio of population in 2020 to the population in 2018. We build a similar model for trucks to predict the number of trucks moving from Jan 1, 2020 to May 7, 2020.

To adjust for changes in car movement due to COVID-19, we use the daily google mobility trends for workplaces. We multiply the projected car movement for 2020 with google mobility to obtain adjusted car movement for 2020. Projected car movement and truck movement for 16 states are shown in Figure 5(a) and Figure 5(b). As part of the essential services to keep the supply chain from breaking, there were no restrictions on the truck movement. Therefore, we do not adjust for changes in truck movement for the period of our study.

**2.5 Train mobility data**

We use Deutsche Bahn’s public timetable to determine all major train routes in Germany. We first identify the 110 biggest cities in Germany and their respective states. We use these major cities to identify movement across states and neighboring countries. Each train station has two timetables (See Figure S6(a) and (b)) for that station – one table shows the arrival time of all the trains to that station (including the departure time from its previous stations) and the other table shows the departure time of all the trains from that station (including the arrival time of its previous stations). For each train, using both the schedules, we find a complete route (based on the 100 biggest cities in Germany). The number of passengers boarding a train for the next station is kept proportional to the sequence of the cities in the train route. We identified 14,712 trains. There are 33 types of trains (based on speed, distance travel and capacity). We assume the total number of passengers in a train to be 400. For long distance trains e.g. ICE, THA, and TGV, we assume the number of passengers in a train to be 600.

Due to COVID-19 and state policies, several trains were canceled, and train movement declined. To account for these changes, we adjust the number of passengers using Google mobility data. We do not actual number of passengers traveling across states from the Deutcshe’s Ban Schedule. We adjust the number of passengers traveling by train after the outbreak of COVID-19 using Google mobility trends at transit stations. Community mobility in transit stations is the change in number of users in and around transit stations as compared to baseline defined by Google.

**2.6 Bus mobility data**

We use travel search history provided by a large third-party European bus and train price comparison and booking company to estimate the number of passengers moving across cities (states) in Germany and passengers traveling to Germany from neighboring countries. The bus data contains the number of searches for a route (departure city to arrival city) aggregated by the day. For example, 33 people searched for buses from Frankfurt to Heidelberg on January 25, 2020. The data does not show the actual number of travelers in the bus, but we use this data as an indicator for bus movement across Germany and its neighboring countries. We only include connections that *arrive* at or *depart from* Germany. We define a bus route as the tuple [departure city, arrival city, date]. The dataset contains the history for 857,159 unique connections (aggregated by day) from December 1, 2019 to May 7, 2020. Of these, 191,356 routes had either their origin or destination in Germany. A subset of 116,706 routes originated and ended in Germany. We assume a capacity of 20 passengers in each bus to estimate the number of travelers. On March 16, 2020, all bus trips were halted in Germany.

**2.7 Flight mobility data**

We use flight transportation information from *https://opensky-network.org*. It is an open source platform containing historical information of all airborne flights. The database uses Automatic Dependent Surveillance – Broadcast (ADS-B) trajectories and maps it with airport International Civil Aviation Organization (ICAO) codes to identify the departure and arrival airport of a flight. The database also maintains the UNIX timestamp for each contact signal (trajectory recorded). We use the *last* UNIX timestamp of a flight to identify its date of arrival and departure. We only consider flights with arrival or departure airports in Germany. We use ICAO airport codes to identify the state for each airport. We ignore flights for which neither the departure nor the arrival airport can be established. The dataset has 187526 flights (with arrival or departure in Germany) for the period of December 1, 2019 to May 7, 2020. The variation in the number of flights over time is shown in Figure S6. We assume a capacity of 200 passengers for a domestic flight and 500 passengers for an international flight to determine the number of individuals on a flight.

**3. Effect of Non-Pharmaceutical Interventions (NPI) on Social Distancing**

To understand the effect of policy interventions on social distancing, we build linear regression model. We use data from February 18, 2020 to April 20, 2020 to estimate the coefficients for different policies. Note that not every policy was implemented by each state as of April 20, 2020, and none of the implemented policies were relaxed until April 20. State governments started relaxing these policies after April 20, 2020. So we use data up to April 20 to estimate the coefficients of different policies. We use Google community mobility ($C_{j,t}$) for retail and recreation to create a measure for deriving social distancing ($sd_{j,t}$) in state $j$ on day $t$. We use smoothed (7-day moving average) social distancing in our linear regression model in Equation S8. Raw and smoothed social distancing numbers for 16 states is shown in Figure S7.

We also use Google Trends, weather data, and general dissatisfaction as additional control variables to account for latent awareness levels in the population, the tendency to leave one’s home due to the higher temperatures during Spring in Germany, and the distress felt due to confining oneself. We consider Google Trends data for the search term “*COVID-19*”. Google Trends is an indicator of search interest of a topic over time. It calculates the proportion of all other searches at the same time and normalizes it to the range of 0 - 100. We normalize the data to 0 – 1 in our model. High Google Trend numbers indicate high interest for the topic during that time. As more cases were observed around the world and Germany, public interest in COVID-19 increased. Increased search is also an indicator for public awareness towards increasing social distancing. Google Trends data for all the sixteen states is shown in Figure S8**.** We use 7 days exponentially smoothed Google Trends in our analysis to account for increased awareness over time. We use exponential smoothing over 7 days to indicate higher awareness through active searching instead of search history. The smoothing function is shown in Equation S7 where $\bar{trend_{j,t}}$ are the raw numbers for Google Trends. The model is robust to changes in the value of the constant $U$ (we use $U=0.8$ in our analysis). The model is robust to changing the smoothing function. Also, we use maximum temperature recorded in a day to account for increased public interest in going out as summer approaches. Maximum Daily temperature for all the sixteen states is shown in Figure S9**.**

$$trend_{jt}=\frac{\sum_{d=1}^{7} U^{7-d}\bar{trend_{j,t}}}{\sum_{d=1}^{7} U^{7-d}} (S7)$$

The linear regression model used to measure the effect of NPIs on mobility $C_{i,t}$ is shown in Equation S7 ($K$ is a constant). We use a binary variable $x_{j,p,t}=1$ if policy $p$ is active in state $j$ on day $t$. ${trend}_{j,t}$ is the Google Trends number for search term in state $j$, and ${temp}_{j,t}$ is the daily maximum temperature in degree Celsius. We use $t$ as index for days. To account for state-level heterogeneity using state fixed effects ($state_{j}$ in Equation S7). $state_{j}$ assumes a value of one if the data considered are specific to that state, and zero otherwise. This allows for control of state-level characteristics that are not in the model and helps reduce the errors due to omitted variables in our analysis. In addition to state-level differences, we also control for week based differences and day of week based differences by incorporating week fixed effects and day of week fixed effects. The variable $week_{t}$ takes a value of 1 if the data considered is in the $w^{th}$ week from February 18, 2020 (For example, all days from February 18, 2020 to February 24, 2020 have $week_{o}= 1$). Variable $day_{t}$ takes a value of 1 if the data considered are specific to that day of the week. Since we consider data for 63 days in our linear regression model, we consider 8 weeks (week 9 is considered as the base week with has 0 fixed effect). For stable parameter estimation, we consider state Thuringia as our base state (0 state fixed effects) and Monday as our base day (0 day of week fixed effect).

$$C_{j,t}=K+\sum_{p=1}^{7} {\beta_{p}^{policy}x}_{j,p,t}+\beta_{t}^{trend}{trend}_{j,t}+\beta_{m}^{temp}{temp}_{j,t}+$$

$$\sum_{j=1}^{16-1} \beta_{j}^{state}state_{j}+\sum_{w=1}^{9-1} \beta_{w}^{week}week_{t}+\sum_{d=1}^{7-1} \beta_{d}^{day}day_{t}+\epsilon_{j,t} (S8)$$

Results for the parameter estimates and summary of the linear regression model is provided in Table S3. The coefficients (and 95% confidence intervals) for different NPIs is given in Figure S10. Predicted and social mobility numbers $({sd}_{i}=-\frac{C_{i}}{100})$ for different states in Germany is shown in Figure S11.

**3.1 Selecting Measure for Social Distancing**

In our analysis, we use community mobility to derive our measure for social distancing $({sd}_{i}=-\frac{C_{i}}{100})$. We select Google community mobility for retail and recreation to create a measure for social distancing. Google community mobility reports collect data from android users. We also collect data for social mobility from Apple for iOS users (Apple’s Community Mobility Report [10]. Apple’s community mobility data includes change in trend in movement as compared to January 13, 2020. The collect data on driving, transit and walking. Apple’s community mobility provides state wise data for Germany only for driving (shown in Figure S12). Correlation between the six measures of community mobility by Google and Apple is shown in Table S4. Community mobility for Retail and Recreation is highly correlated with all the other indicators of community mobility (with Pearson correlation > 0.5 for grocery and pharmacy, transit stations, workplaces and residential in Google community movement data and Driving in Apple community movement data). Since it is highly correlated with all other indicators of community mobility expect Google community mobility for Parks. However, few people visited parks during the pandemic. Hence, we consider Community mobility for Retail and Recreation as a measure for social distancing. Note that the community mobility provided by Google and Apple community movement reports do not include actual movement data (or actual community mobility). It is an indicator to changes in trend in the movement as compared to their respective baselines.

**3.2 Robustness Check: Lasso Regression Model**

Lasso regression places a penalty on the sum over the absolute value of coefficients. As some of the NPIs were introduced at the same time, this could lead to multicollinearity in NPIs. We use a penalized regression model (Equation S9) to check the robustness of the estimate from linear regression model in Equation S7 ($|l|$= magnitude of scalar $l$). We use 5-fold cross validation to estimate the parameter coefficients using Lasso regression. The estimates are shown in Table S5. Robustness check using Lasso shows that the parameter estimation from Linear regression model in Equation S8 is robust.

$$C_{j,t}=K+\sum_{p=1}^{7} {\beta_{p}^{policy}x}_{j,p,t}+\beta_{t}^{trend}{trend}_{j,t}+\beta_{m}^{temp}{temp}_{j,t}+$$

$$\sum_{j=1}^{16-1} \beta_{j}^{state}state_{j}+\sum_{w=1}^{9-1} \beta_{w}^{week}week_{t}+\sum_{d=1}^{7-1} \beta_{d}^{day}day_{t} +\lambda\left( \left( \sum_{p=1}^{7} |\beta_{p}^{policy}| \right)+{|\beta}^{trend}\left| +{|\beta}^{temp} \right|+\left( \sum_{j=1}^{16-1} |\beta_{j}^{state}| \right)+\left( \sum_{d=1}^{7-1} |\beta_{d}^{day}| \right)+\left( \sum_{w=1}^{9-1} |\beta_{w}^{week}| \right) \right) +\epsilon_{j,t} (S9)$$

Predicted and Google Community mobility numbers $C_{i}$ for different states in Germany is shown in Table S5. Lasso regression shrinks the coefficients to 0 such that only significant predictor variables effect the prediction. Therefore, Lasso regression does not provide confidence interval on the estimates of the coefficients. Also, estimates of confidence interval for weighted linear regression models are biased, so we do not provide confidence intervals for Lasso regression. However, to obtain the Interquartile range (IQR) for the parameter estimates, we adopt a bootstrapping method. In bootstrapping, we run our Lasso regression model 10,000 times with 90% of the data and obtain the estimates for the coefficients. We calculate IQR from the parameter estimates of these 10,000 runs. Results for the parameter estimates from the Lasso regression model is provided in Table S5.

**3.3 Robustness Check: Potential Spillover from Neighboring States**

Within the SEIR model, we already account for spillovers between states by implementing most interstate movement (car, flight, bus, train, and truck). Furthermore, spillovers will also be accounted for by Google Trends, as people in a state would be more cautious and search for “COVID-19” on the internet more frequently if cases are rising in neighboring states. Moreover, we now include state fixed effects to account for state level heterogeneity. This new specification may also help alleviate potential spillover of a particular state from its neighbors.

Social distancing may be influenced by control variables outside of those considered in our focal model (Equation S8). We test two alternative model specifications to check the robustness of the parameter estimation. In the first alternative specification (R1), we use social distancing numbers from neighboring states to account for spillovers from other states (Equation S10). We only consider states in Germany for neighboring states. $NE_{j}$ is the number of neighbors for state $j$ and $j(ne)$ represents the $ne^{th}$ neighbor of state $j$.

$$C_{j,t}=K+\sum_{p=1}^{7} {\beta_{p}^{policy}x}_{j,p,t}+\beta_{t}^{trend}{trend}_{j,t}+\beta_{m}^{temp}{temp}_{j,t}+$$

$$\sum_{j=1}^{16-1} \beta_{j}^{state}state_{j}+\sum_{w=1}^{9-1} \beta_{w}^{week}week_{t}+\sum_{d=1}^{7-1} \beta_{d}^{day}day_{t}+\sum_{ne=1}^{NE_{j}} \beta_{ne}^{neighbor}sd_{j,t-1}+\epsilon_{j,t} (S10)$$

In the second model (R2), we use social distancing from a day before as a covariate in our model as shown in Equation S11. It controls for awareness in the previous day (i.e., if an individual did not go out due to COVID-19 yesterday, they will also not go out today due to COVID-19).

$$C_{j,t}=K+\sum_{p=1}^{7} {\beta_{p}^{policy}x}_{j,p,t}+\beta_{t}^{trend}{trend}_{j,t}+\beta_{m}^{temp}{temp}_{j,t}+$$

$$\sum_{j=1}^{16-1} \beta_{j}^{state}state_{j}+\sum_{w=1}^{9-1} \beta_{w}^{week}week_{t}+\sum_{d=1}^{7-1} \beta_{d}^{day}day_{t}+\beta^{yesterday}sd_{j,t-1}+\epsilon_{j,t} (S11)$$

The coefficients of the focal model and both alternative specifications R1 and R2 are shown in Figure S13. We find that the coefficient estimates of all three models are close to one another. Given that there may be more covariates that could affect social mobility and are not included in our model, we cannot claim causality. However, these coefficients still provide significant insights into the potential of an NPI to induce social distancing in the community.

**3.4 Leaving Out One Policy at a Time**

NPIs were implemented across states in different sequence (Figure 1 in Manuscript). However, the sequence they were implemented were not random, hence we cannot claim causality on the impact of an individual (using coefficients from linear regression models) NPI on social distancing. To check the robustness of the estimation of coefficients from the linear regression model, we simulate different scenarios when a particular policy (randomly selected) was never introduced in one of the state (randomly selected). Results for the parameter estimates from 10,000 simulations is shown in Figure S14. This simulation is designed to strongly bias the estimate. It assumes that one randomly chosen NPI did not occur, while the population’s mobility changes in that state was kept as actually observed in the data. In a robust model specification, we hope that the parameter estimates remain similar to the original ones even though these strong biases are introduced. Results in Figure S14 show that the coefficients from the linear regression simulation by leaving our one policy in a state (where it was implemented) are close to the coefficients estimated from our focal model in Equation S8. We find that the estimated (biased) coefficients are comparable to the ones of the focal model and conclude that our model is robust to minor random changes in the sequencing on NPIs

**4. Generating Different What-If Scenarios**

To understand the contribution of different policies in containing the spread of COVID-19, we consider different sets of scenarios for which policies were implemented and relaxed. State governments started introducing different policies around Mid-March. They started relaxing some of these policies on April 20, 2020 (Figure 3). We use data from Feb 18, 2020 to April 20, 2020. We create test scenarios with all the NPIs – border closures, educational activities closure, contact restriction order lifted, initial businesses opened, stay at home orders lifted, non-essential services opened, retail outlets opened, and all policies in place.

Figure 3 shows the timeline for policy introduction across different states in Germany. Some states did not introduce all the seven policies and in some cases introduced certain policies on varying dates, allowing for a quasi-experimental set-up to test the effect of policies on social distancing and spread of disease subsequently. Next, we create eight counterfactual scenarios (one for each policy and one for all policies in place) to study what will happen if the states lift the focal policy.

Different states across Germany started relaxing some policies on Apr 20, 2020. To determine the effect of lifting a policy we examine policy relaxation in two scenarios: What would have happened if the policies were relaxed on April 21 or April 28, by easing one policy at a time to estimate the marginal effect of a policy. The week-long delay helps determine the increase in cases by relaxing a policy one week earlier.

**4.1 Delay in NPI Relaxation**

The differential effects allow for rank-ordering the policies by order of their impacts on disease spread. If a policy was relaxed on April 21 (without changing other policies), the social distancing would decrease after April 21. Similarly, if a policy was relaxed on April 28 (without changing other policies), social distancing will start decreasing after April 28 (without changing policies from April 21 to April 27 and keeping it as it is). In this analysis, we simulate scenarios when the policies are relaxed (one at a time) on either 21 April, 2020 or April 28, 2020 to understand the impact of delay in reopening. We predict mobility (hence social distancing) for 90 days from April 20, 2020. The social distancing under different scenarios for different states are shown in Figure S15 and Figure S16**.**

We use Krinsky-Robb method [9] to estimate confidence intervals around the predictions. Krinsky-Robb method takes advantage of the assumption that coefficients of linear regression follow a multivariate normal distribution. Krinsky method then uses Cholesky decomposition of the covariance matrix of the coefficients to get random draws from multivariate normal distribution. Monte Carlo simulation draws are used to get the confidence interval across the predictions (predicted on different simulation draws). In our analysis, we use 10,000 random draws to estimate the confidence bounds for the predictions. Note that we use week fixed effects in our linear regression model which is not available when projecting in the future. When generating scenarios, we predict based on 0 fixed effects for week when predicting from April 21 onwards.

**4.2 Mobility Bounce Back after NPI Relaxation**

In this work, we postulate that introduction of NPIs led to increase in social distancing that helped in containing the spread of the virus. However, NPIs cannot be implemented indefinitely and must be relaxed at some point in time. Due to increased awareness over time, we expect that mobility will not go back to normal (0 $C_{j,t})$ at least in the near future (or right after a pandemic peak). Thus, we expect the social distancing to be greater than 0 even when all the NPIs are relaxed. To verify potential bounce back in community mobility, we predict community mobility if all the policies were relaxed on April 20, 2020. Social distancing for all the 16 states under all policies relaxed on April 20, 2020 is shown in Figure S18. It shows that even though social distancing decreases sharply when all the NPIs are relaxed, social distancing does not go down to 0 immediately i.e., community mobility does not bounce back to baseline levels. This is due to other covariates included in the model in Equation S8 e.g. Google Trends, maximum daily temperature and various fixed effects. This verifies that even when all the policies are relaxed, mobility does not go back to normal immediately when all policies are relaxed.

**5. Effective Reproduction Number**

The effective reproduction number $R_{e}$ is the average number of new infected cases caused by a single infected case on a given day. The effective reproduction number $R_{e}$ for the SEIR compartmental model can be calculated with the method introduced in [7] and later expanded in [8]. The value of $R_{e}$ changes by an increase or decrease in social mobility of both the infected and susceptible population. Other factors impacting the value of $R_{e}$ include environmental conditions and the drop in susceptible population over time.

In the SEIR model, $X=[E, I^{d},I^{u}]$ are considered the infected compartments of the model. $R_{e}$ can be calculated as the leading Eigen value of the next generation matrix (NGM), $K=\mathrm{FV}^{-1}$. $F=\frac{\partial\mathcal{F}_{i}(x)}{\partial X_{j}}$ is the Jacobian matrix of the rate of new infections in infected compartments and $V=\frac{\partial\mathcal{V}_{i}(x)}{\partial X_{j}}$ is the Jacobian matrix of the rate of transitions between infected compartments. Using equations (S1-S4), we have:

$\mathcal{F=}\left[ \begin{matrix} \frac{\left( 1-\gamma*sd \right)\beta SI^{d}}{N}+\frac{\left( 1-\gamma*sd \right)\mu\beta SI^{u}}{N} \\ 0 \\ 0 \end{matrix} \right]$, $\mathcal{V=}\left[ \begin{matrix} \frac{E}{Z} \\ \frac{I^{d}}{D}-\frac{\alpha E}{Z} \\ \frac{I^{u}}{D}-\frac{(1-\alpha)E}{Z} \end{matrix} \right]$ (S12)

$F= \left[ \begin{matrix} 0 & \frac{\left( 1-\gamma*sd \right)\beta S}{N} & \frac{\left( 1-\gamma*sd \right)\mu\beta S}{N} \\ 0 & 0 & 0 \\ 0 & 0 & 0 \end{matrix} \right]$, $V= \left[ \begin{matrix} \frac{1}{Z} & 0 & 0 \\ -\frac{\alpha}{Z} & \frac{1}{D} & 0 \\ -\frac{1-\alpha}{Z} & 0 & \frac{1}{D} \end{matrix} \right]$ (S13)

And calculating $R_{e}$:

$R_{e}=(\left( 1-\gamma*sd \right)\alpha\beta D+\left( 1-\gamma*sd \right)(1-\alpha)\mu\beta D)\frac{S}{N}$ (S14)

Figure S17 shows the effective reproduction number over time in different states of Germany using inferred parameters values. These values are in-line with the reported values for Germany 0.79 [95%CI: 0.66 – 0.90] using nowcasting approach as a moving 4-day average by Robert Koch Institute [2]. As depicted in this figure, the reproduction number at the beginning of the epidemic in Germany is above 1 in all states. Once the social distancing policies are in effect, this rate drops to below 1 in all states. When $R_{e}>1$, the disease starts to spread throughout the population. Equation S14 shows that $R_{e}$ also decreases with a drop in the susceptible fraction of population. A large proportion of population must transition from susceptible to infected, immune, or dead for this factor to be considerable.

To understand the effects of social distancing on $R_{e}$, we simulate the effect of lifting different policies on the value of $R_{e}$. Different social distancing polices are simulated to be removed on April 21, 2020 and April 28, 2020. Removing some policies would increase $R_{e}$ to above 1 in some states. For example, in large states such as Baden-Wurttemberg (BW) and Bayern (BY), $R_{e}$ will rise above 1 if the contact restriction order is lifted or initial businesses are opened. Certain policies must be kept in effect until the susceptible proportion of population drops significantly for $R_{e}$ to remain below 1.

The dependency of $R_{e}$ to different combinations of parameters $\alpha$, $\gamma$, and $sd$ is depicted in Figure S19. These figures represent the basic reproduction number where all population is assumed to be susceptible. Figure S19 (a) represents the changes in $R_{e}$ with respect to possible values of parameters $\alpha$ and $\gamma$. High $\alpha$ and low $\gamma$ combinations result in the highest $R_{e}$ value. Figure S19 (b) shows that $R_{e}$ monotonically increases with decrease in $sd$ and increase in $\alpha$. Figure S19 (c) shows that $R_{e}$ will increase in combinations of high $\gamma$ and low $sd$, while its value drops with high $\gamma$ and high $sd$ combinations. As $\gamma$ decreases, the dependency of $R_{e}$ to $sd$ also declines.

Figure S20 shows the ratio of undocumented cases to daily infected cases across Germany from our model in Equation S1-S4. Undocumented case from the model can also be used to estimate effective reproductivity number.

**Figures and Tables**


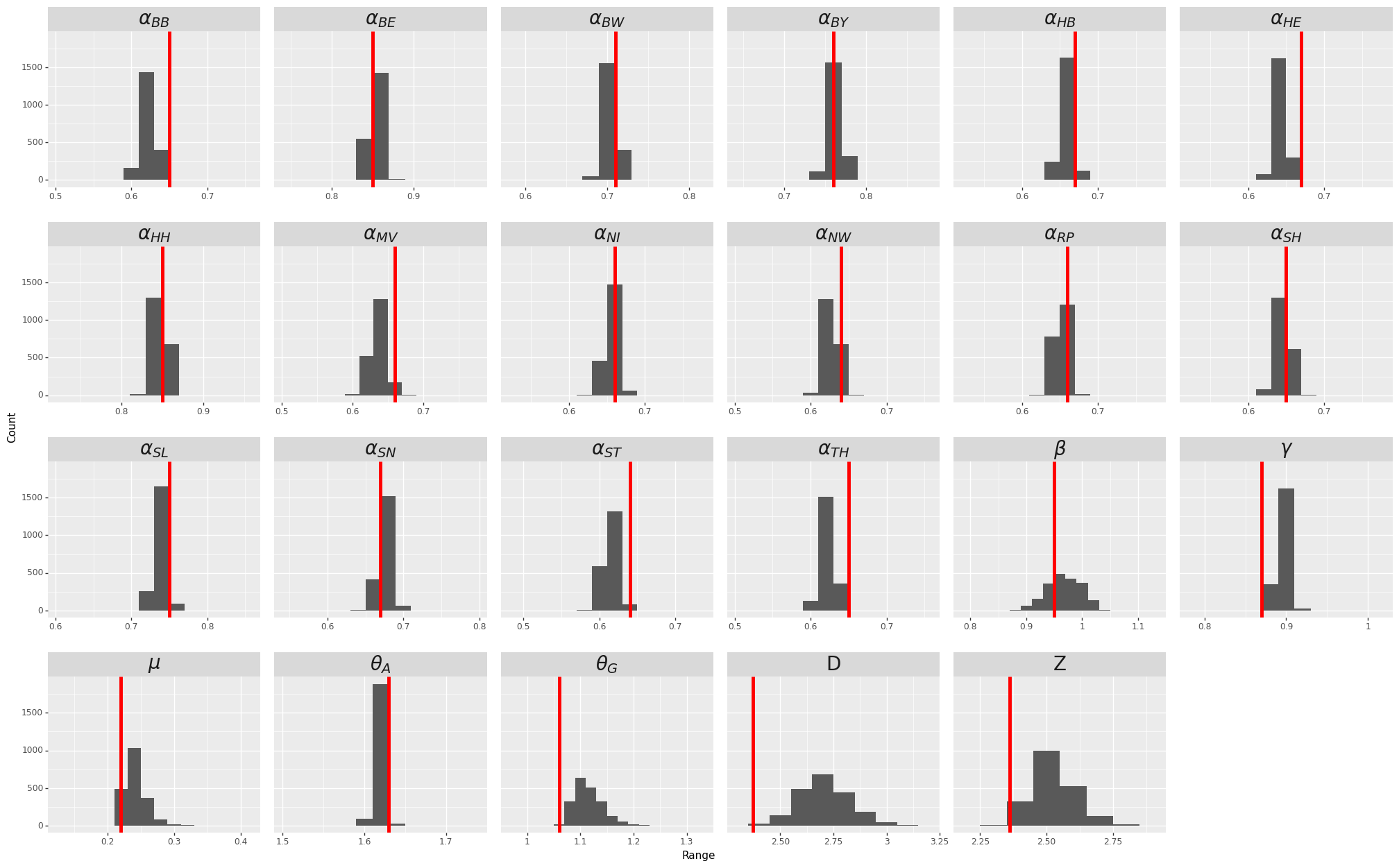


***Figure S1.*** *Model parameter inference. Initial day for the model is set to Feb 18, 2020. Histograms show distribution of model parameters inference over 2000 runs, each with 500 ensembles. Each run provides a set of parameters as a group that is optimized together and should be used as one. Red lines show the set of inferred parameters of a randomly selected run used for estimation.*

| 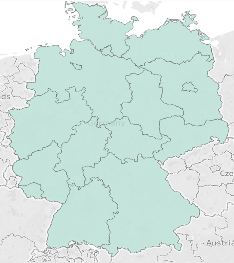(a) February 22  Total Cases: 14 | 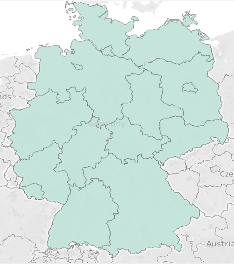(b) February 29  Total Cases: 66 | 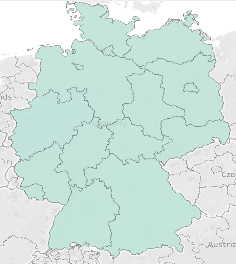(c) March 7  Total Cases: 684 | 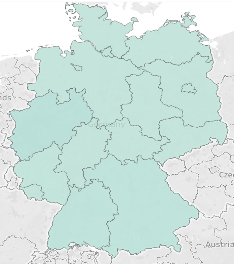(d) March 14  Total Cases: 14 | 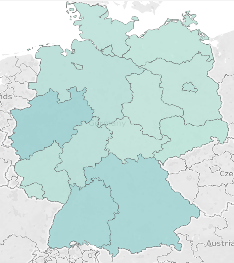(e) March 21  Total Cases: 3795 | 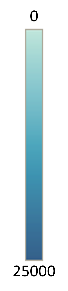 |
| --- | --- | --- | --- | --- | --- |
| 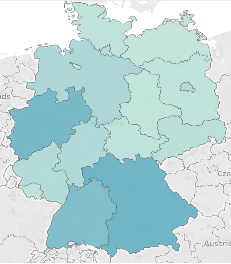(f) March 28  Total Cases: 16662 | 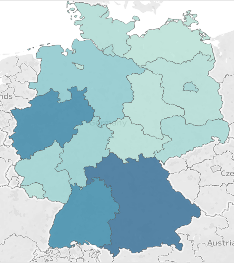(g) April 4  Total Cases: 85778 | 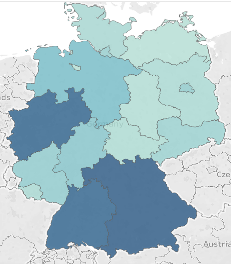(h) April 11  Total Cases: 117658 | 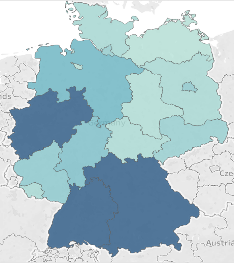(i) April 18  Total Cases: 137439 | 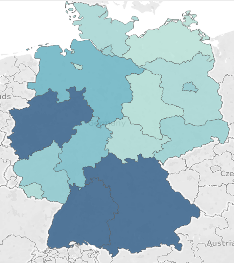(j) April 25  Total Cases: 152438 |  |

***Figure S2****. Spread of COVID-19 across Germany over time (Figures generated using Heat Map feature in Tableau Software, Version 2020.2 [11]).*


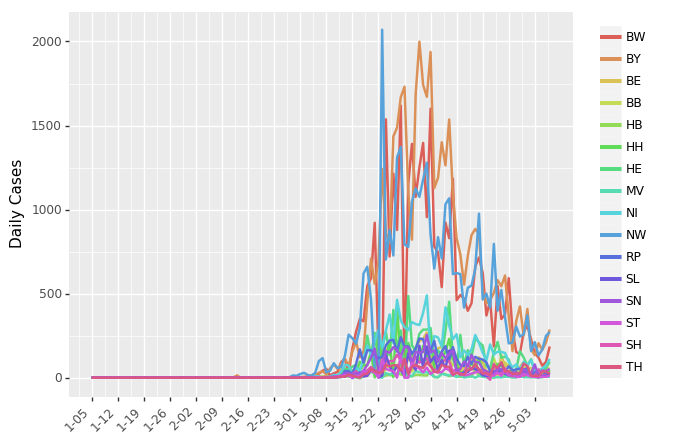


***Figure S3.*** *Daily New Cases from RKI (Adjusted for Midnight Reporting)*


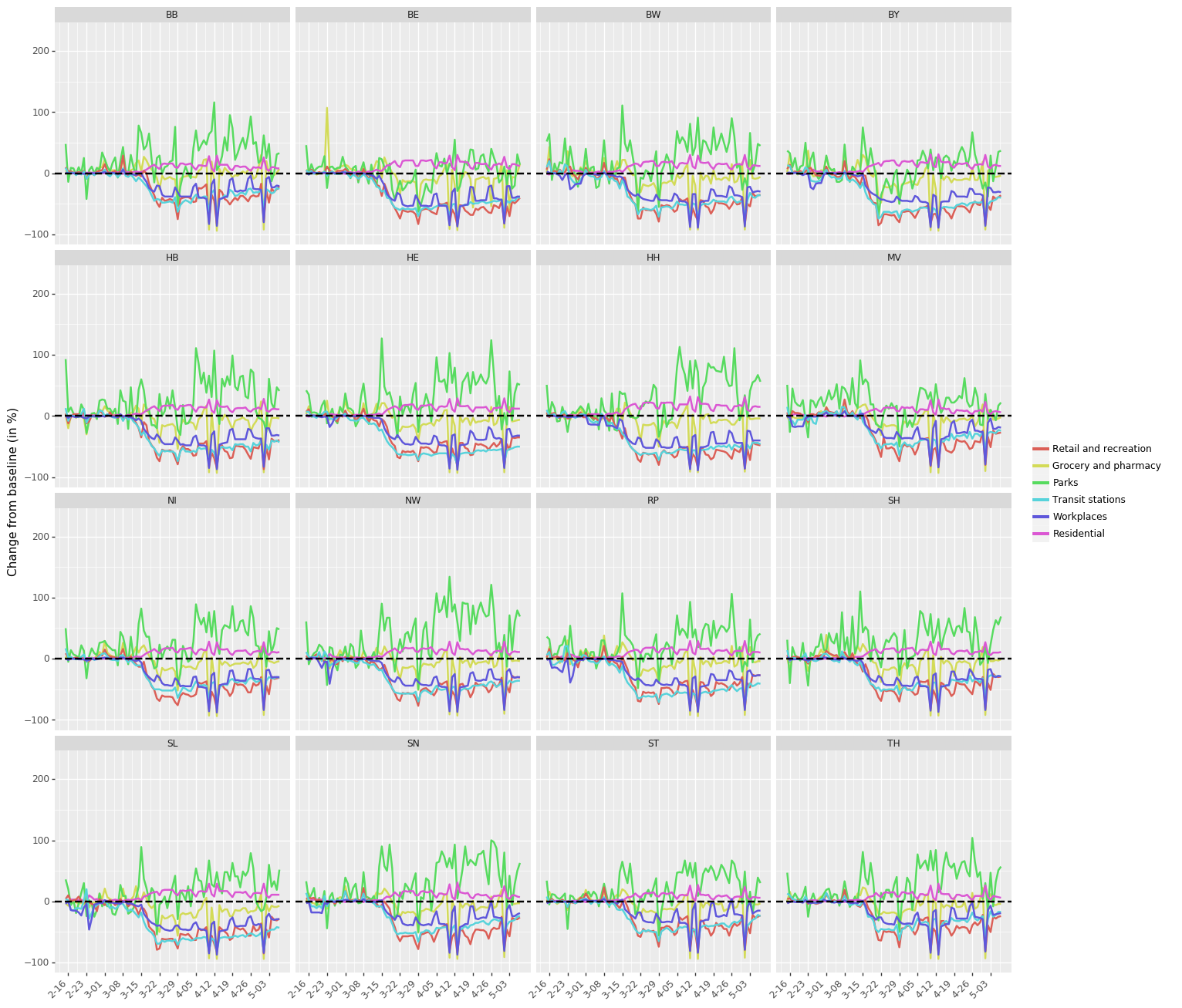
***Figure S4.*** *Google Community Mobility*

| 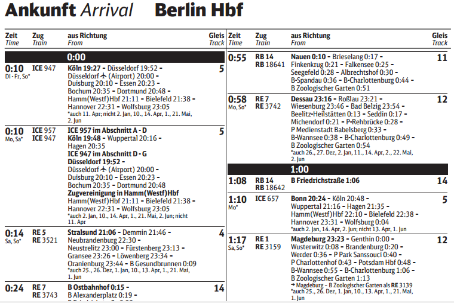 | 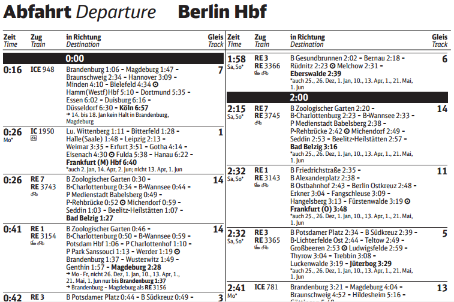 |
| --- | --- |

***Figure S5.*** *Ground transportation using trains in Germany. Panel (a) shows part of the train timetable available in all German train stations. More specifically, the sample snippet displayed in Panel (a) lists the departure times of trains leaving Berlin Hbf. We parse 538 timetable files to obtain the train schedule for all of Germany. We combine information from the arrival and departure timetables to construct the complete route of a train. Panel (b) lists the departure time for all the trains from Berlin with arrival time of all the succeeding stations for that train. We combine information from Panel (a) and Panel (b) to construct the complete route of a train.*


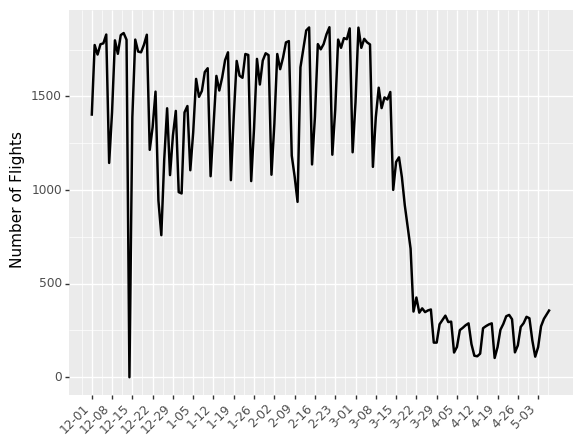


***Figure S6.*** *Daily Flights Arriving to Different Airports in Germany*


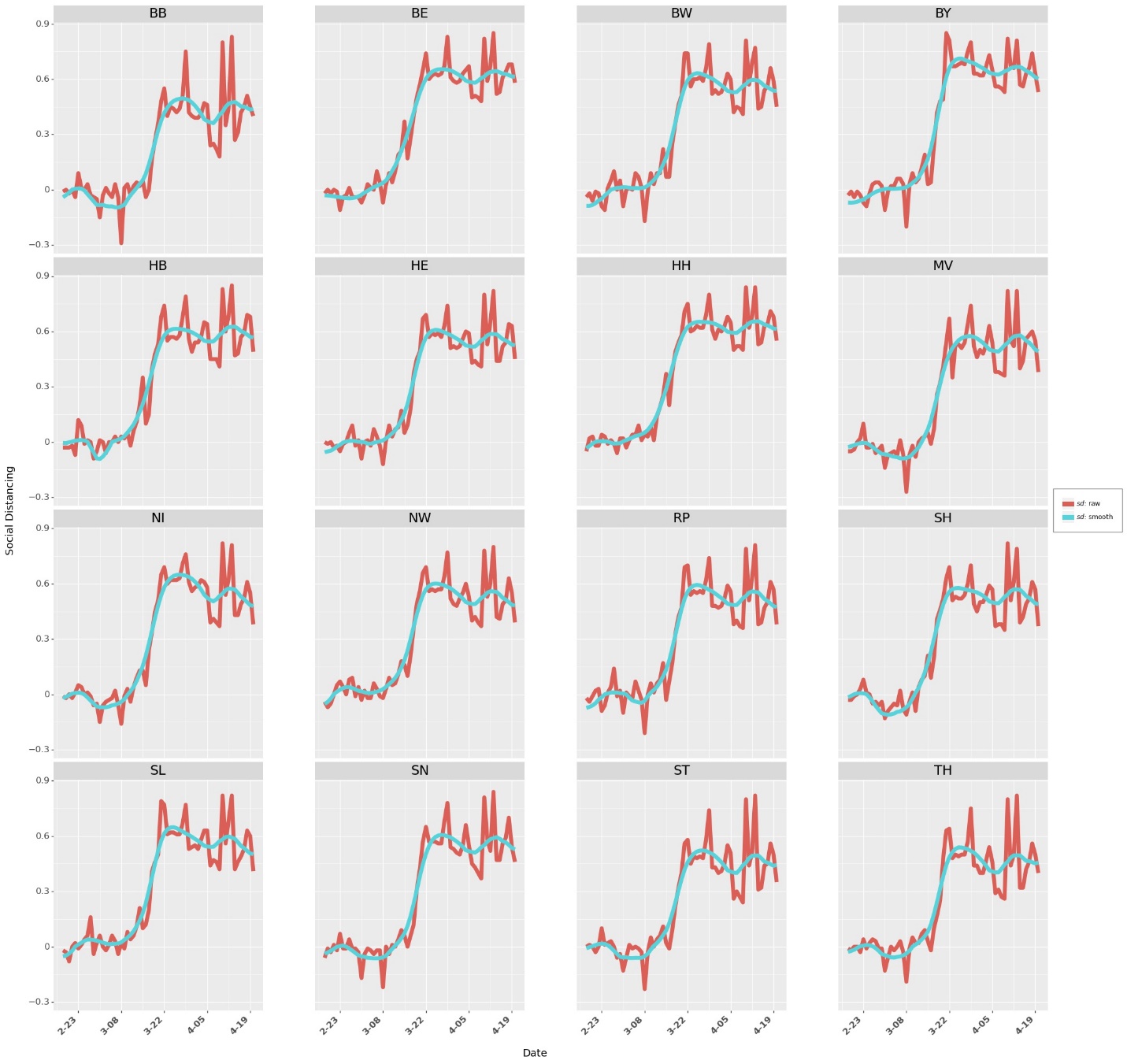


***Figure S7.*** *Raw and Smoothed Social Distancing in Different States*


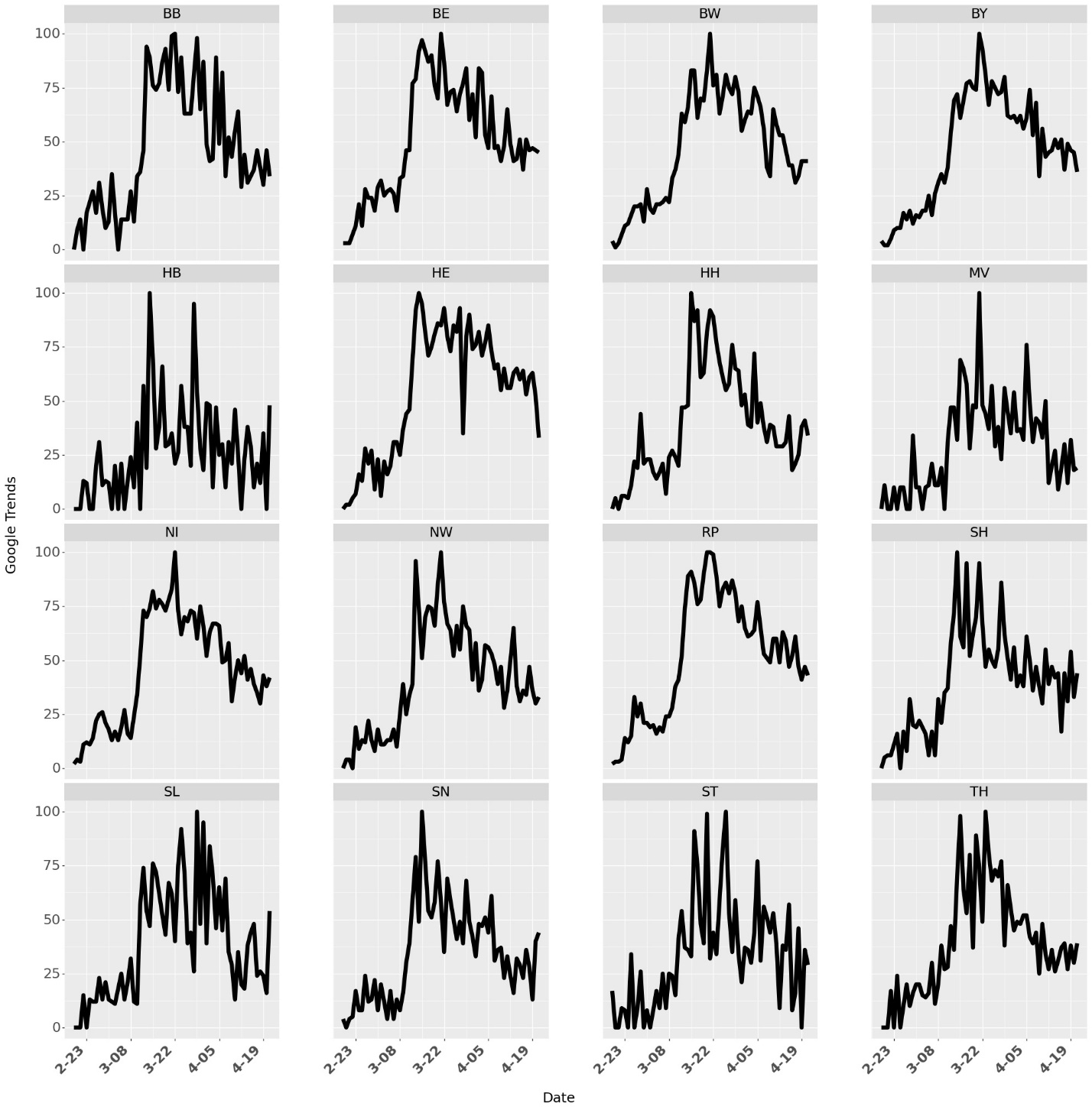


***Figure S8.*** *Google Trends Data for Different States in Germany*


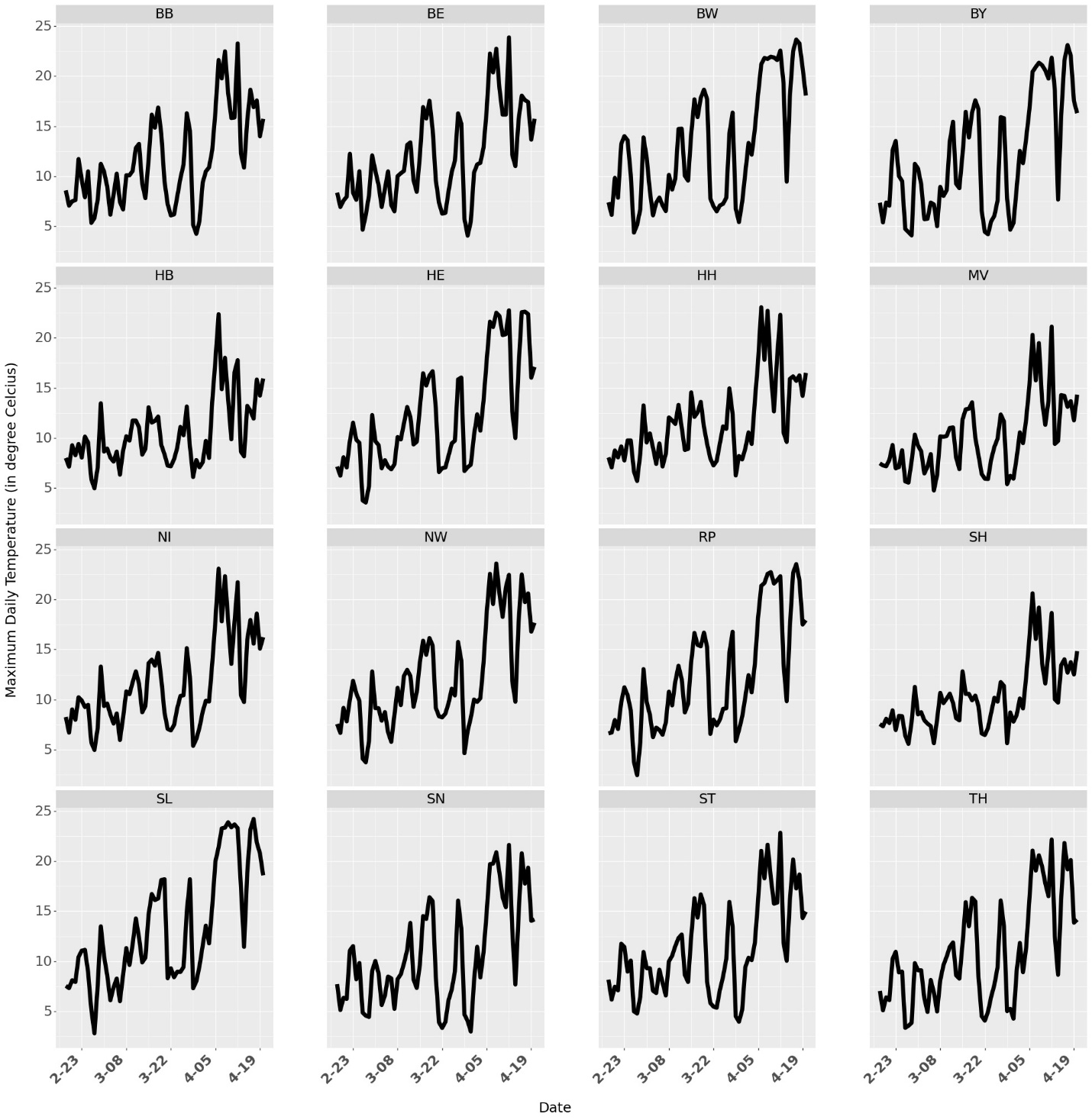


***Figure S9.*** *Maximum Daily Temperature Data for Different States in Germany*

***
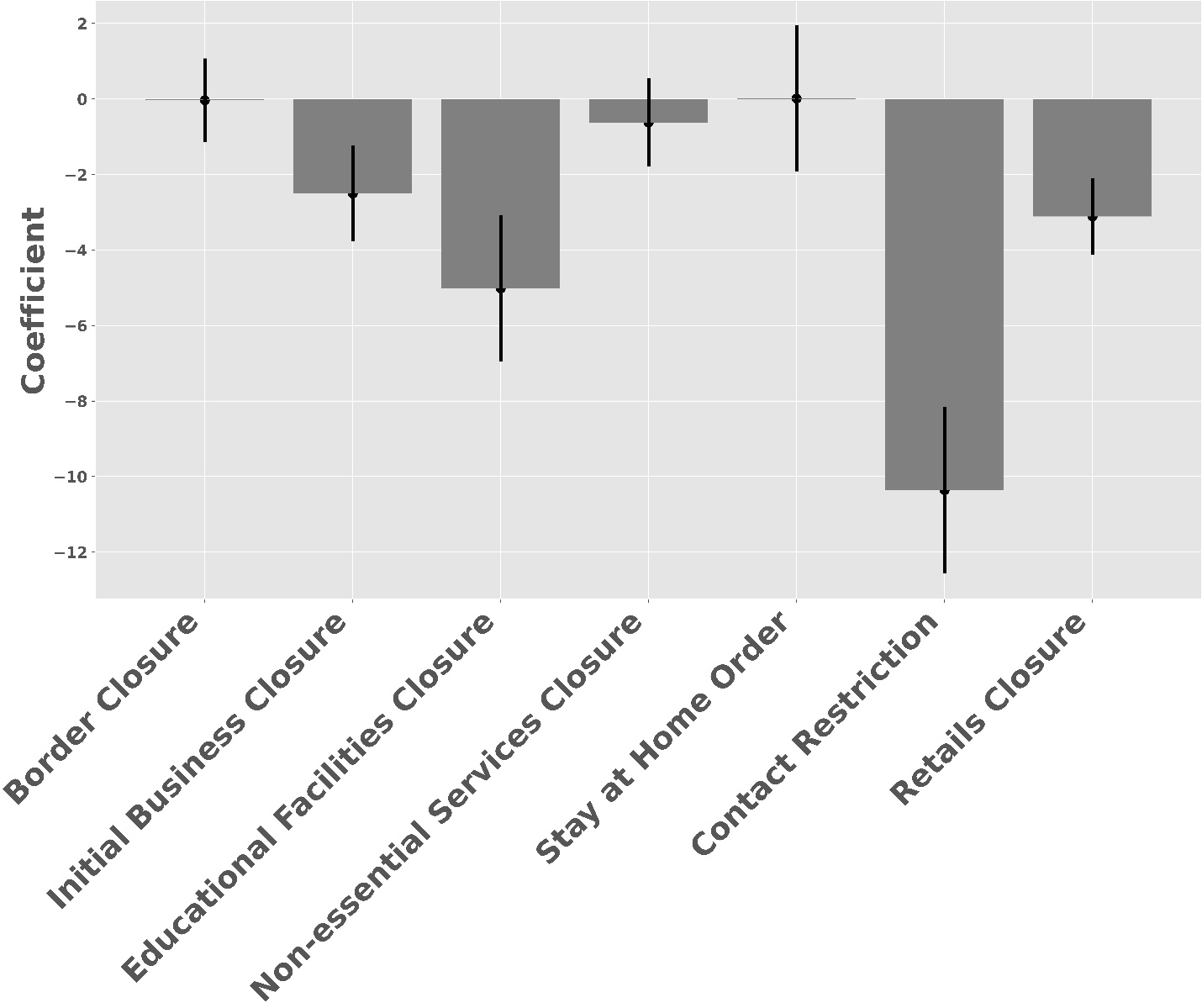
***

***Figure S10.*** *Parameter Estimates for Different NPIs. Vertical grey lines represent the 95% confidence interval for the estimates.*

| 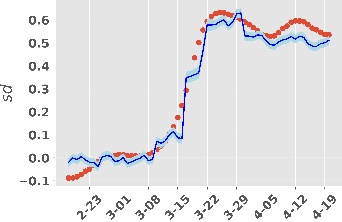  (a) BW | 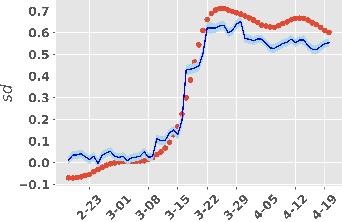  (b) BY | 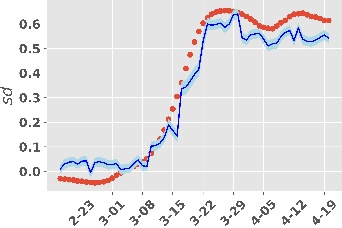  (c) BE | 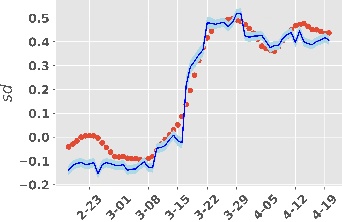  (d) BB |
| --- | --- | --- | --- |
| 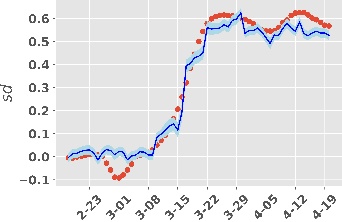  (e) HB | 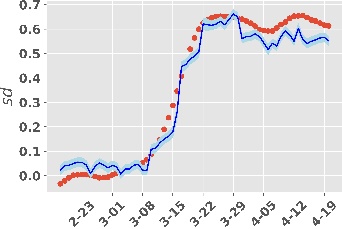  (f) HH | 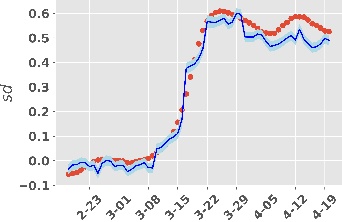  (g) HE | 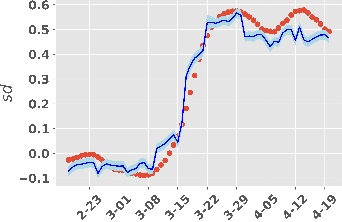  (h) MV |
| 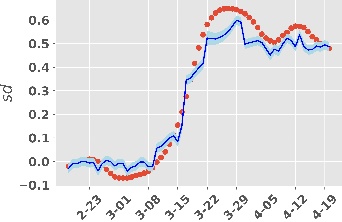  (i) NI | 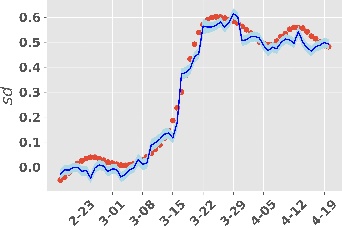  (j) NW | 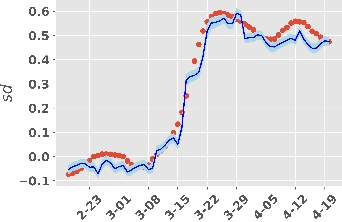  (k) RP | 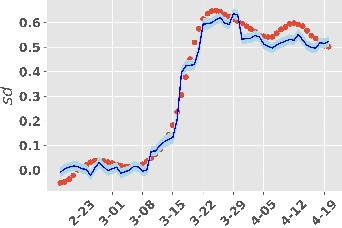  (l) SL |
| 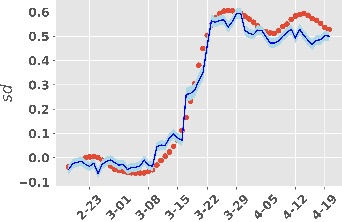  (m) SN | 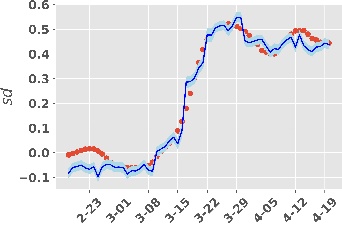  (n) ST | 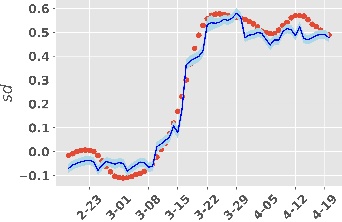  (o) SH | 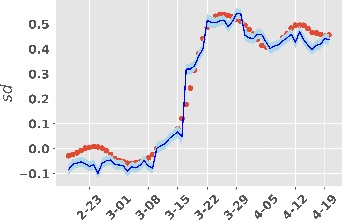  (p) TH |

***Figure S11.*** *Predicting Social Distancing for Different States in Germany. Red Dots shows smoothed social distancing. Solid blue line shows the estimate for predicted* $sd$*. Light blue shade is the confidence intervals around the predicted* $sd$*. The model achieves an adjusted r-square value of 0.978.*

***
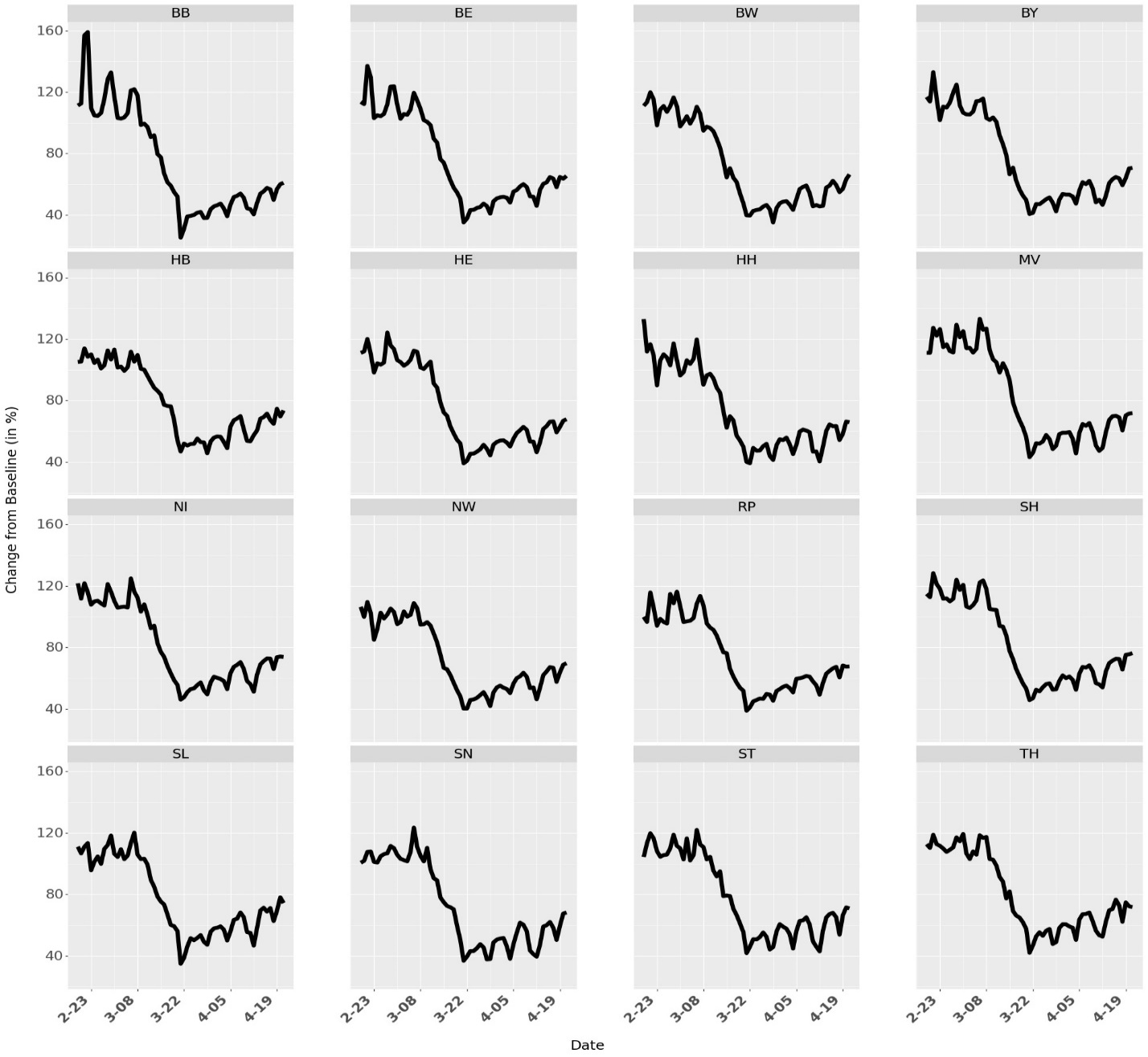
Figure S12.*** *Apple Community Mobility (Driving)*

*
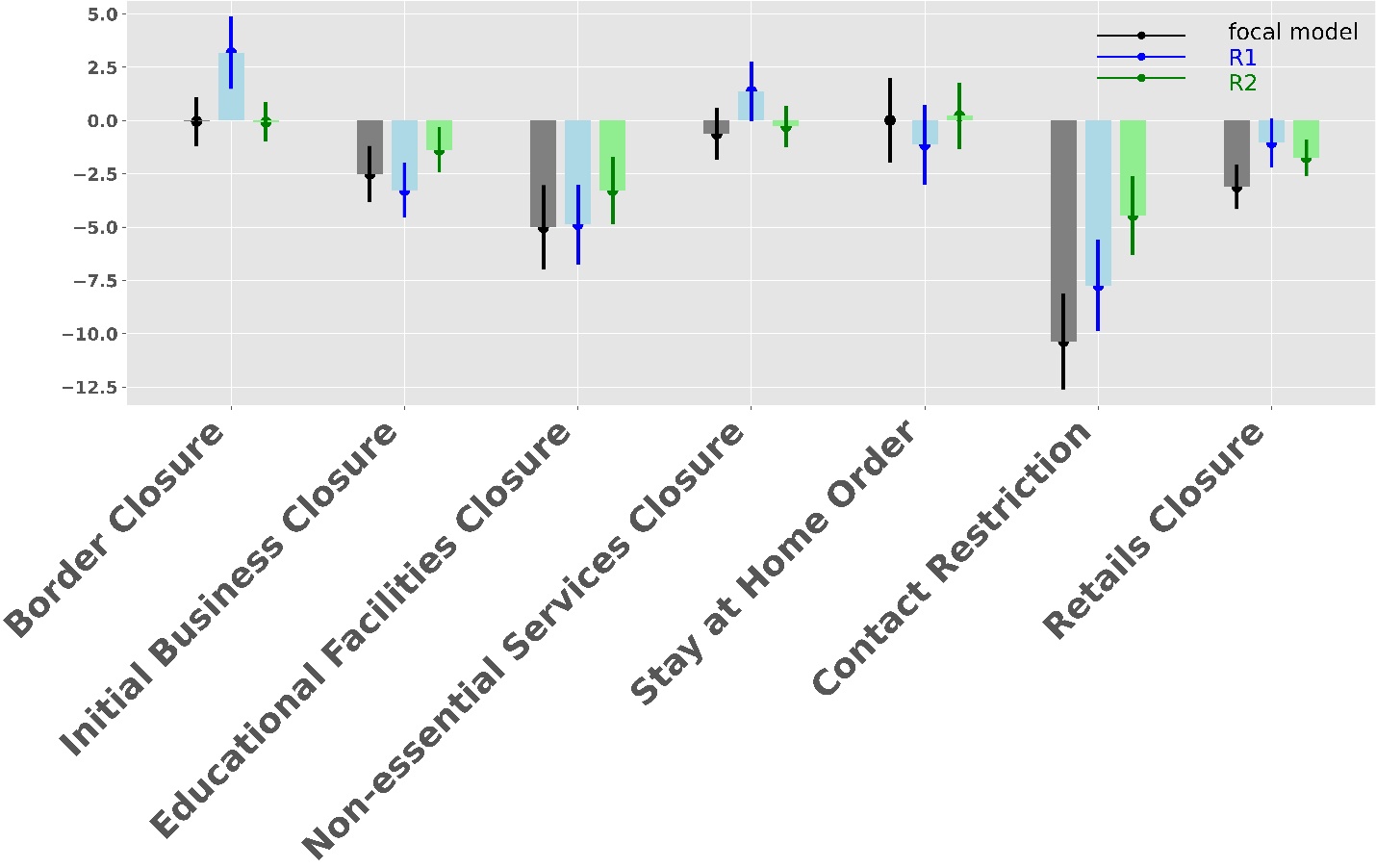
*

***Figure S13.*** *Parameter Estimates from Two Robustness Check Models R1 and R2 in Section 3.3.*

*
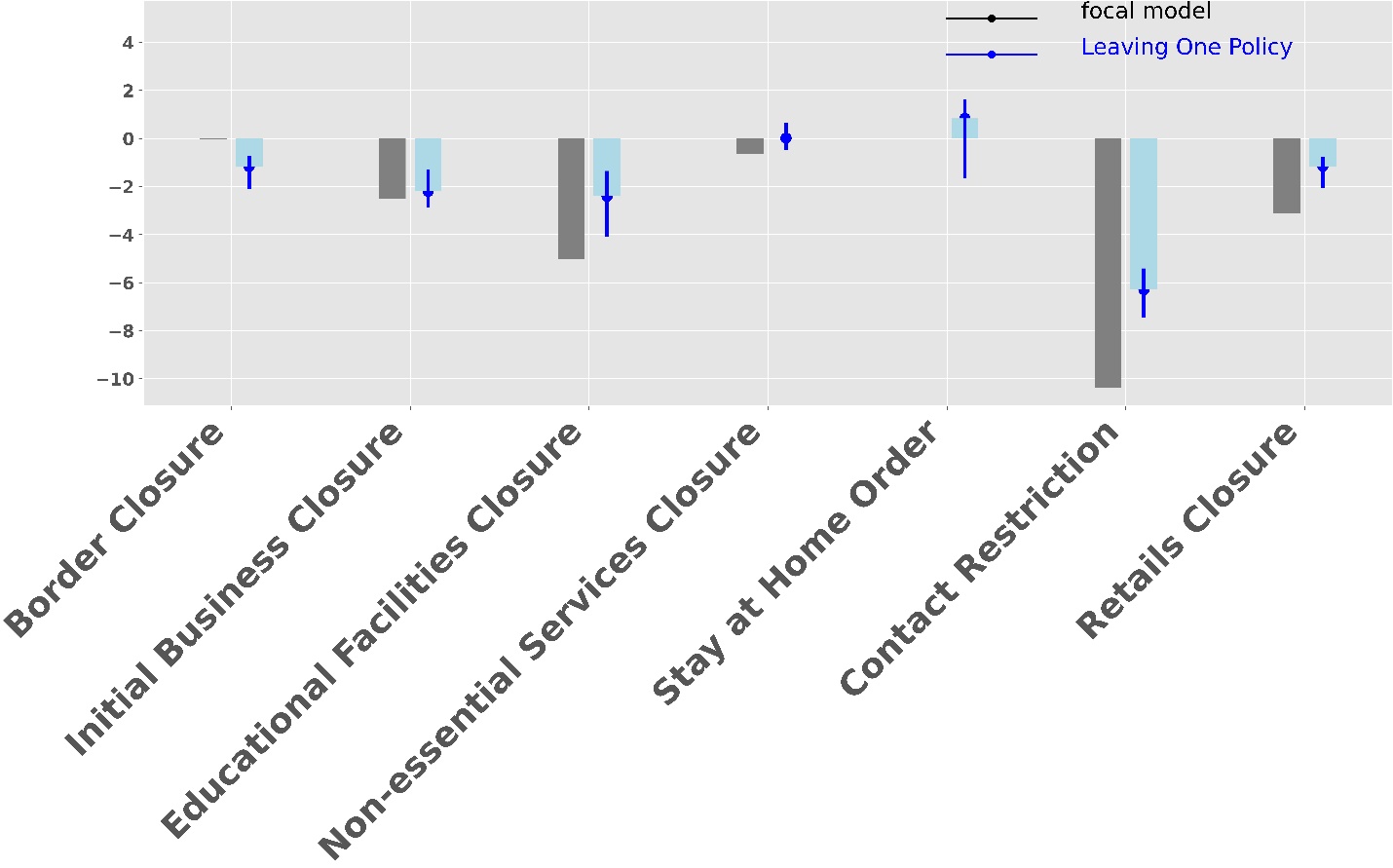
*

***Figure S14.*** *Parameter Estimates from Linear Regression when One NPI is never Implemented in one of the states (one state and one NPI selected randomly in one simulation). The grey bar shows the coefficients from linear regression. Green bars show the 50^th^ percentile of coefficients from the simulation. Vertical green lines show the 25^th^ and 75^th^ percentile of the coefficients from the simulation.*

*
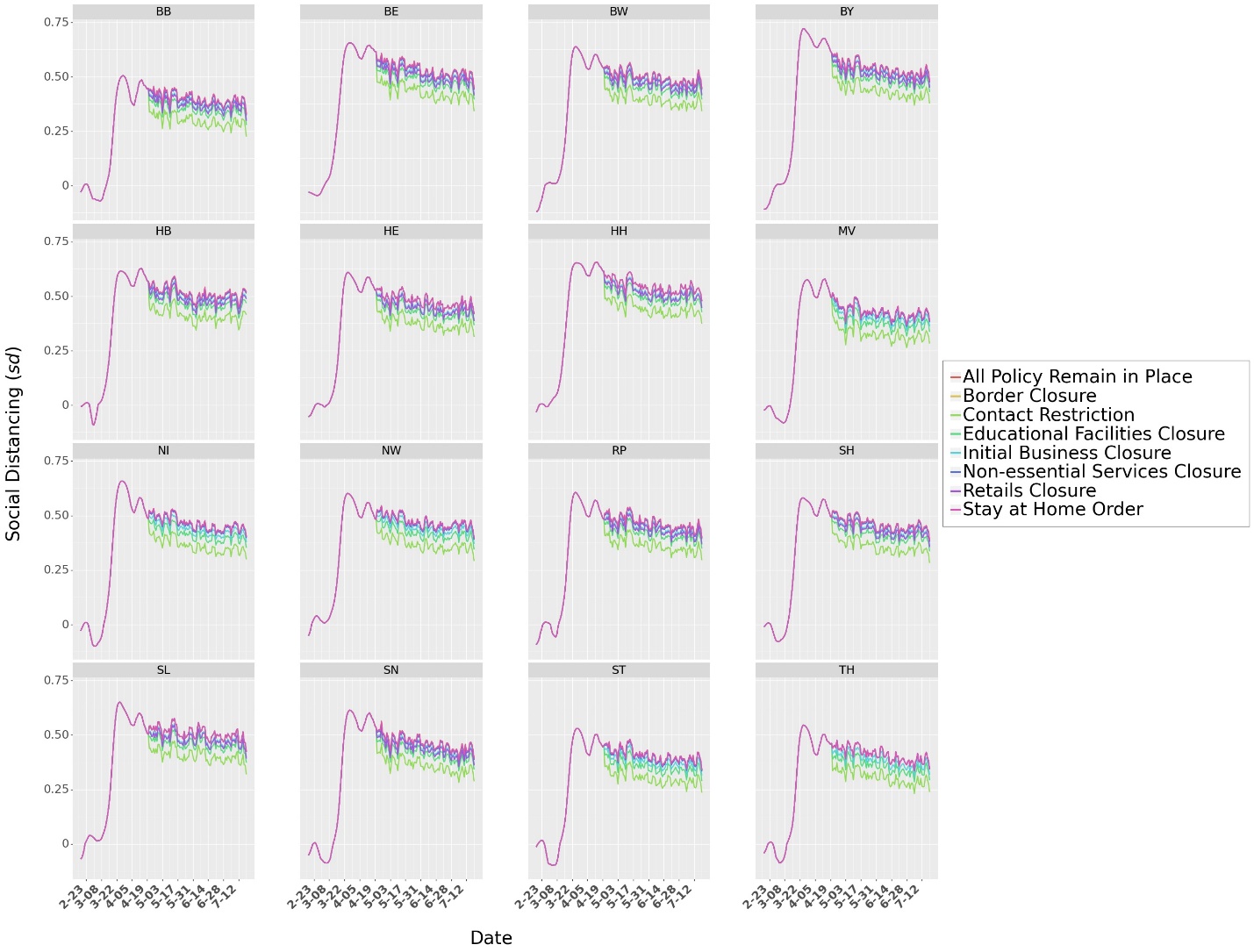
*

***Figure S15.*** *Scenario 1: Social distancing* $sd_{i}$*when policies are relaxed on April 21, 2020*

*
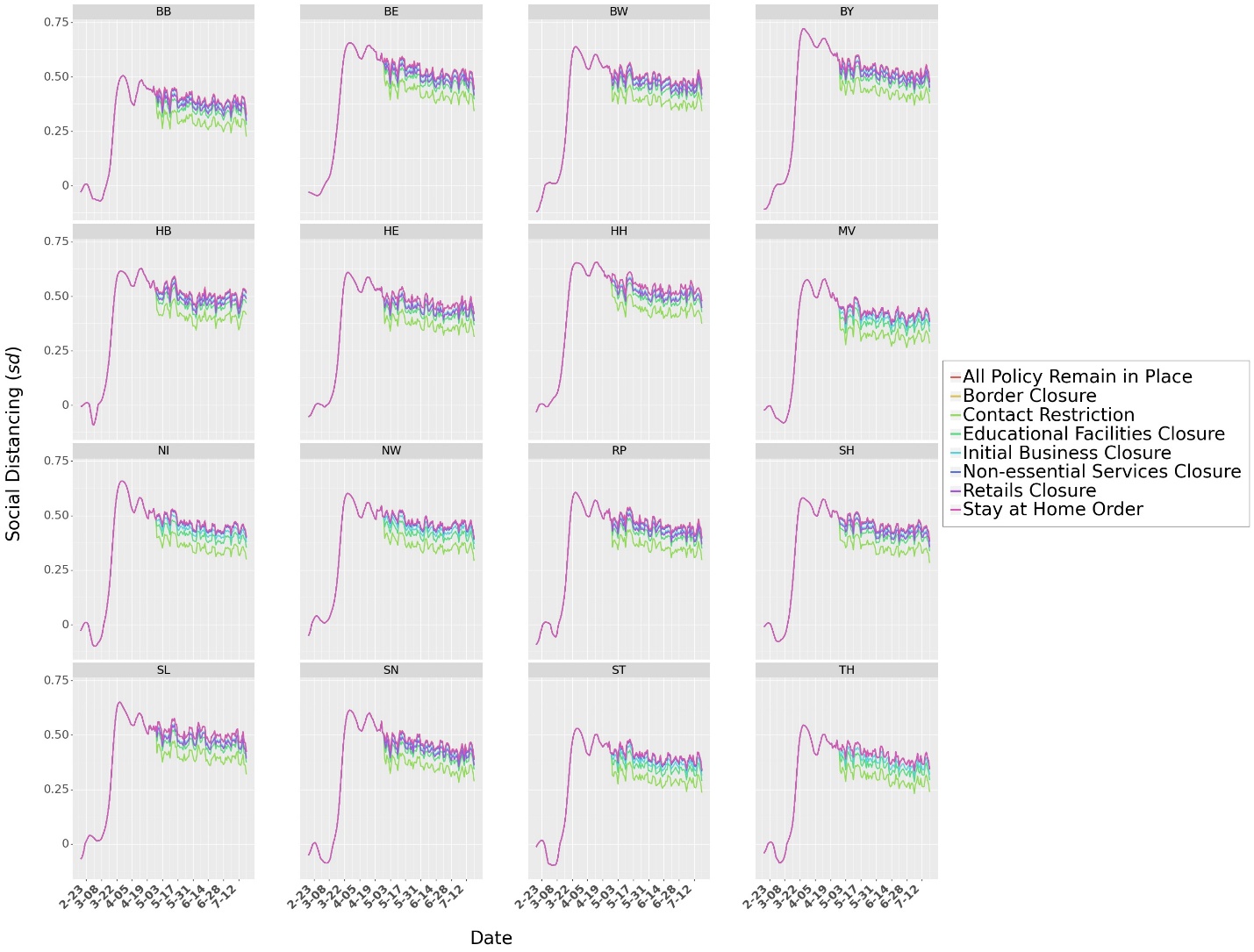
*

***Figure S16.*** *Scenario 2: Social distancing* $sd_{i}$*when policies are relaxed on April 28, 2020*


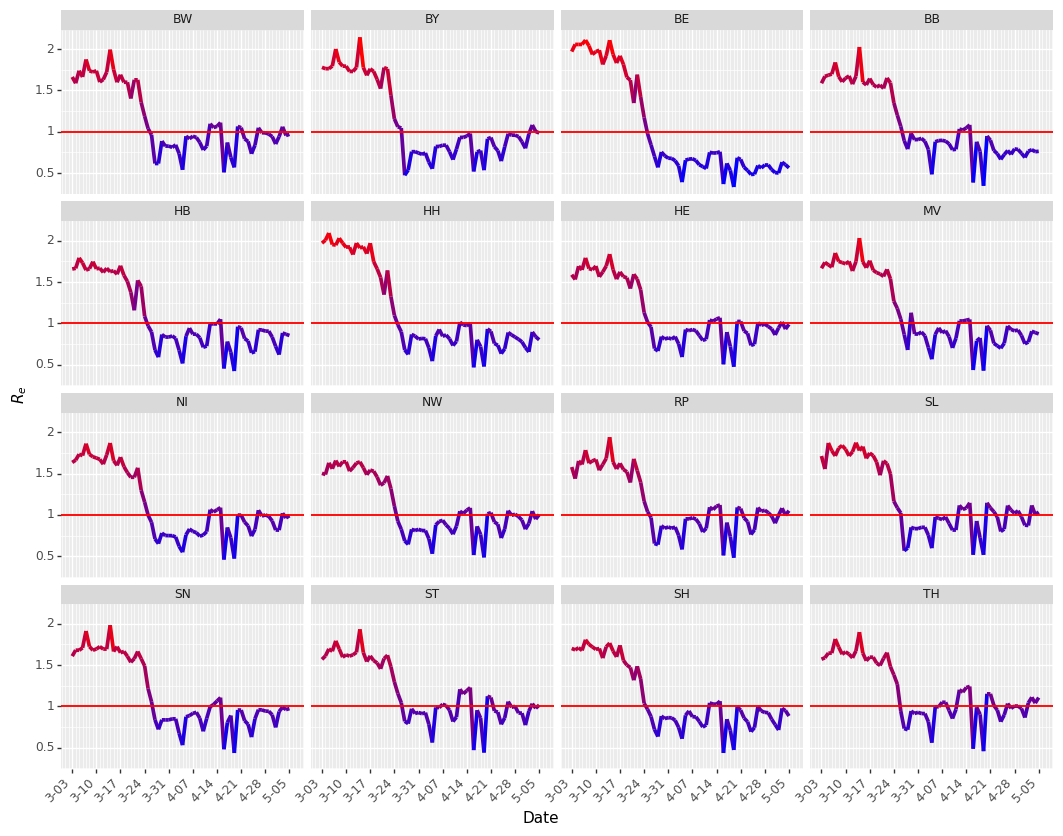


***Figure S17.*** *Effective reproduction number. The values of* $R_{e}$ *drops from above the red line 1 to below 1 after policies are in effect.*

| 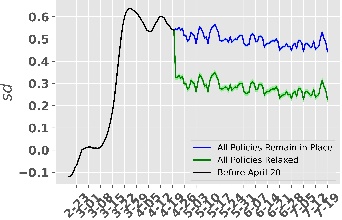  (a) BW | 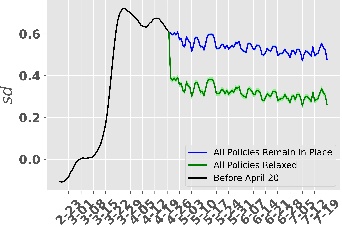  (b) BY | 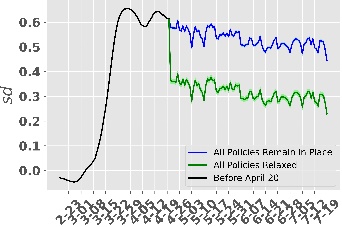  (c) BE | 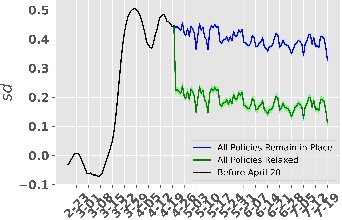  (d) BB |
| --- | --- | --- | --- |
| 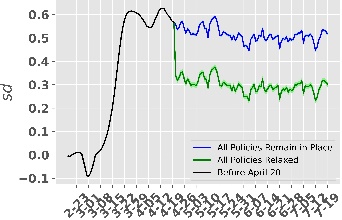  (e) HB | 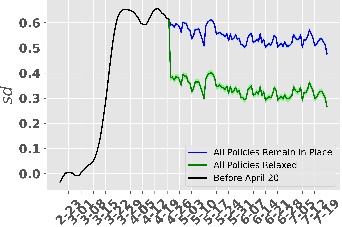  (f) HH | 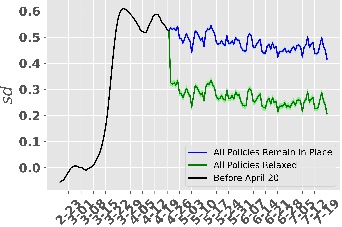  (g) HE | 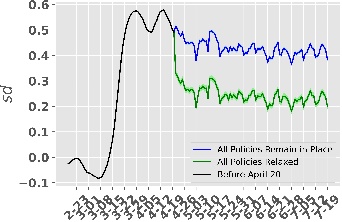  (h) MV |
| 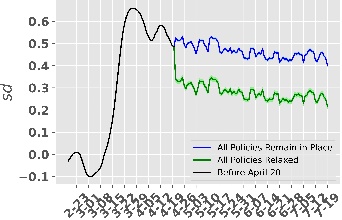  (i) NI | 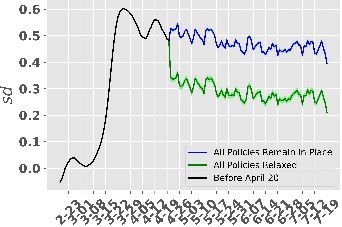  (j) NW | 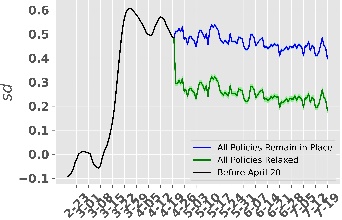  (k) RP | 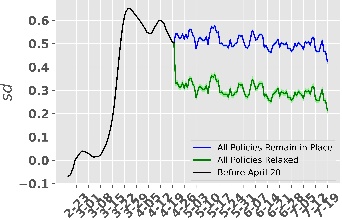  (l) SL |
| 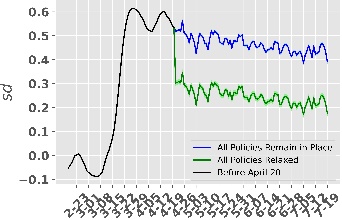  (m) SN | 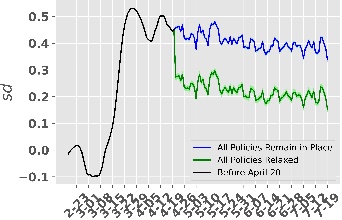  (n) ST | 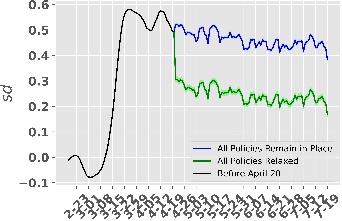  (o) SH | 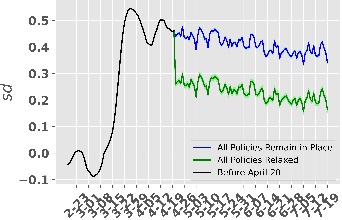  (p) TH |

***Figure S18.*** *Predicting Social distancing under different Scenarios. Green line shows the predicted social distancing when all the policies are relaxed on April 20, 2020. Blue line shows the predicted social distancing when all the policies remain in place till July 19, 2020. Black line shows actual social distancing numbers till April 20, 2020, derived from community mobility reports.*


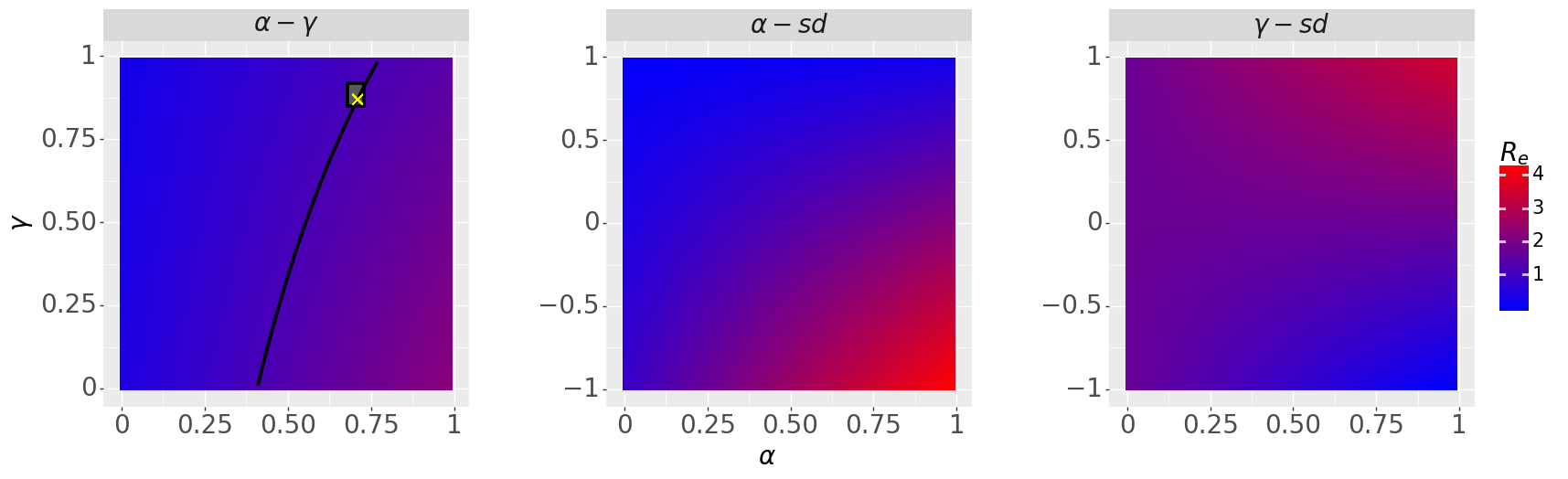


**a**

**c**

**b**

γ

α

*sd*

*sd*

***Figure S19.*** *The dependency of* $R_{e}$*on model parameters* $\alpha$ *and* $\gamma$ *and social mobility* $sd$*. All other variables are held constant with values represented in Table S1 for state BW.* $\alpha-\gamma$*: The impact of* $\alpha$ *and* $\gamma$ *on* $R_{e}$*. The value of sd is set to* $sd= 0.35$*. Black curve represents the constant* $R_{e}=1.21$*. The box represents the interval of inferred parameters that have the maximum likelihood and the yellow ‘X’ mark shows the parameter combination used for estimation (*$\alpha=0.71$ *,*$\gamma=0.89$*).* $\alpha-sd$*: The impact of* $\alpha$ *and social mobility* $sd$*on* $R_{e}$*. Note that* $sd$ *is not a model parameter.* $\gamma-sd$*: The impact of* $\gamma$ *and social mobility* $sd$ *on* $R_{e}$*.*

**
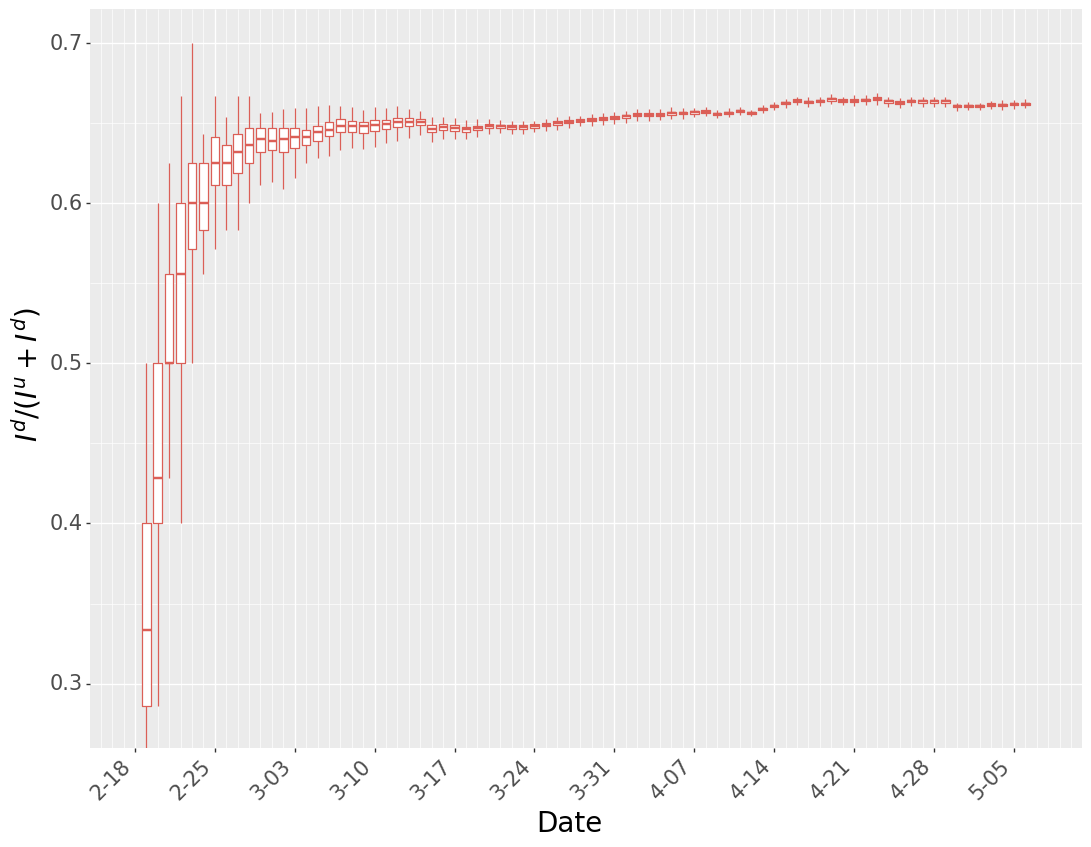
**

***Figure S20****. The ratio of documented infected cases to total daily infected cases for Germany.*

| **Parameters** | **Initial Range** | **Inferred Value [95% Confidence Intervals]** |
| --- | --- | --- |
| $\boldsymbol{\beta}$ | 0.8-1.2 | 0.95 [0.92 – 1.01] |
| $\boldsymbol{\mu}$ | 0.2-1.0 | 0.22 [0.22 – 0.27] |
| $\boldsymbol{\theta}_{\boldsymbol{G}}$ | 1.0-1.8 | 1.08 [1.08 – 1.16] |
| $\boldsymbol{\theta}_{\boldsymbol{A}}$ | 1.0-1.8 | 1.63 [1.61 – 1.63] |
| $\boldsymbol{Z}$ | 2.0-5.0 | 2.41 [2.41 – 2.67] |
| $\boldsymbol{\gamma}$ | 0.6-1.0 | 0.89 [0.89 – 0.91] |
| $\boldsymbol{D}$ | 2.0-5.0 | 2.52 [2.52 – 2.91] |
| $\boldsymbol{\alpha}_{\boldsymbol{i}}$ | 0.5-1.0 | (Per State Estimate Follows) |
| $\boldsymbol{\alpha}_{\boldsymbol{BW}}$ | | 0.71 [0.69 – 0.72] |
| $\boldsymbol{\alpha}_{\boldsymbol{BY}}$ | | 0.76 [0.75 – 0.77] |
| $\boldsymbol{\alpha}_{\boldsymbol{BE}}$ | | 0.85 [0.84 – 0.87] |
| $\boldsymbol{\alpha}_{\boldsymbol{BB}}$ | | 0.64 [0.61 – 0.64] |
| $\boldsymbol{\alpha}_{\boldsymbol{HB}}$ | | 0.67 [0.65 – 0.67] |
| $\boldsymbol{\alpha}_{\boldsymbol{HH}}$ | | 0.85 [0.84 – 0.86] |
| $\boldsymbol{\alpha}_{\boldsymbol{HE}}$ | | 0.65 [0.63 – 0.65] |
| $\boldsymbol{\alpha}_{\boldsymbol{MV}}$ | | 0.65 [0.62 – 0.65] |
| $\boldsymbol{\alpha}_{\boldsymbol{NI}}$ | | 0.66 [0.64 – 0.67] |
| $\boldsymbol{\alpha}_{\boldsymbol{NW}}$ | | 0.64 [0.61 – 0.64] |
| $\boldsymbol{\alpha}_{\boldsymbol{RP}}$ | | 0.66 [0.64 – 0.66] |
| $\boldsymbol{\alpha}_{\boldsymbol{SL}}$ | | 0.75 [0.73 – 0.75] |
| $\boldsymbol{\alpha}_{\boldsymbol{SN}}$ | | 0.67 [0.66 – 0.69] |
| $\boldsymbol{\alpha}_{\boldsymbol{ST}}$ | | 0.63 [0.6 – 0.63] |
| $\boldsymbol{\alpha}_{\boldsymbol{SH}}$ | | 0.65 [0.63 – 0.66] |
| $\boldsymbol{\alpha}_{\boldsymbol{TH}}$ | | 0.64 [0.61 – 0.64] |

***Table S1.*** *Model parameters inference and prior range. The initial range is the prior range for parameters in EAKF algorithm. The selected set of inferred values along with 95% confidence interval is reported.*

| **Policy** | **Description** | **Coefficients** |
| --- | --- | --- |
| Border Closure | Closure of International Borders. Border between Germany and Netherlands were never closed. | $\beta_{1}^{policy}$ |
| Initial Business Closure | Closure of dance events, trade fairs, exhibitions, special markets, arcades, casinos, betting shops and similar companies; amusement places; prostitution institutions, concert halls, fairs, leisure and animal parks, providers of leisure activities and similar facilities | $\beta_{2}^{policy}$ |
| Educational Facilities Closure | Closure of All Educational Facilities, K-12 and University | $\beta_{3}^{policy}$ |
| Non-Essential Services Closure | Closure of bars, clubs, cinemas, theatres, museums, florists, garages, fashion stores and churches. | $\beta_{4}^{policy}$ |
| Stay at Home Order | Residents asked to shelter-in-place | $\beta_{5}^{policy}$ |
| Contact Restriction | Gatherings limited to no more than 5 people, unless family | $\beta_{6}^{policy}$ |
| Retail Outlets Closure | Does not apply to retail for food, weekly markets, pick-up and delivery services, beverage markets, pharmacies, medical supply stores, drug stores, petrol stations, banks and savings banks, post offices, hairdressers, dry cleaners, Laundromats, newspaper sales, DIY and garden centers, pet supplies and wholesale, craftsmen and craft like trades. | $\beta_{7}^{policy}$ |

***Table S2.*** *Description of different NPIs*

| **Model Summary** | | | |
| --- | --- | --- | --- |
| Number of Observations | | 1008 | |
| Degree of Freedom (Residuals) | | 969 | |
| Degree of Freedom (Model) | | 38 | |
| R-square Value | | 0.978 | |
| **Coefficient Estimates** | | | |
| **Variables** | **Lower Confidence Bound** | **Estimate** | **Upper Confidence Bound** |
| Constant ($C$) | -29.559 | -25.6761 *** | -21.793 |
| Border Closure ($\beta_{1}^{policcy})$ | -1.099 | -0.0343 | 1.03 |
| Initial Business Closure ($\beta_{2}^{policy}$) | -3.736 | -2.5041 *** | -1.272 |
| Educational Facilities Closure ($\beta_{3}^{policy}$) | -6.91 | -5.0164 *** | -3.123 |
| Non-essential Service Closure ($\beta_{4}^{policy}$) | -1.753 | -0.6261 | 0.501 |
| Stay at Home Order $(\beta_{5}^{policy}$) | -1.873 | 0.0142 | 1.901 |
| Contact Restriction $(\beta_{6}^{policy}$) | -12.519 | -10.3598 *** | -8.2 |
| Retail Outlets Closure $(\beta_{7}^{policy}$) | -4.076 | -3.1127 *** | -2.149 |
| Google Trends $(\beta^{trend}$) | -4.356 | -3.797 *** | -3.238 |
| Tmax $(\beta^{temp}$) | 0.366 | 0.4473 *** | 0.529 |
| Baden-wurttemberg ($state_{1}$) | -7.389 | -5.7414 *** | -4.094 |
| Bayern ($state_{2}$) | -9.972 | -8.358 *** | -6.744 |
| Berlin ($state_{3}$) | -11.089 | -9.5171 *** | -7.945 |
| Brandenburg ($state_{4}$) | 1.258 | 2.9328 *** | 4.608 |
| Bremen ($state_{5}$) | -7.969 | -6.2889 *** | -4.608 |
| Hamburg ($state_{6}$) | -13.172 | -11.6518 *** | -10.131 |
| Hessen ($state_{7}$) | -5.092 | -3.5518 *** | -2.012 |
| Mecklenburg-Vorpommern ($state_{8}$) | -3.879 | -2.284 *** | -0.689 |
| Niedersachsen ($state_{9}$) | -6.538 | -5.0077 *** | -3.477 |
| Nordrhein-Westfalen ($state_{10}$) | -6.396 | -4.8454 *** | -3.295 |
| Rheinland-Pfalz ($state_{11}$) | -4.333 | -2.6913 *** | -1.05 |
| Saarland ($state_{12}$) | -8.94 | -7.3416 *** | -5.743 |
| Sachsen ($state_{13}$) | -3.631 | -1.9973 ** | -0.363 |
| Sachsen-anhalt ($state_{14}$) | -0.77 | 0.7554 | 2.281 |
| Schleswig-holstein ($state_{15}$) | -2.55 | -0.9342 | 0.681 |
| Week 0 ($week_{o})$ | 24.024 | 27.3614 *** | 30.699 |
| Week 1 ($week_{1})$ | 27.835 | 31.0584 *** | 34.282 |
| Week 2 ($week_{2})$ | 29.941 | 33.1193 *** | 36.298 |
| Week 3 ($week_{3})$ | 22.275 | 25.1858 *** | 28.097 |
| Week 4 ($week_{4})$ | 7.409 | 9.7545 *** | 12.1 |
| Week 5 ($week_{5})$ | 3.094 | 4.7599 *** | 6.426 |
| Week 6 ($week_{6})$ | 5.694 | 7.0033 *** | 8.312 |
| Week 7 ($week_{7})$ | 0.018 | 1.1626 ** | 2.307 |
| Tuesday ($day_{1}$) | 1.98 | 2.9742 *** | 3.969 |
| Wednesday ($day_{2})$ | 0.78 | 1.7576 *** | 2.735 |
| Thursday ($day_{3})$ | -0.152 | 0.8219 * | 1.795 |
| Friday ($day_{4})$ | -0.541 | 0.4362 | 1.414 |
| Saturday ($day_{5})$ | -1.477 | -0.5065 | 0.464 |
| Sunday ($day_{6})$ | -0.909 | 0.0431 | 0.995 |
| *** p<0.01, ** p<0.05, * p<0.1, | | | |

***Table S3.*** *Linear Regression Summary*

|  | Google:  Retail and Recreation | Google:  Grocery and Pharmacy | Google:  Parks | Google:  Transit Stations | Google:  Workplace | Google:  Residential | Apple:  Driving |
| --- | --- | --- | --- | --- | --- | --- | --- |
| Google:  Retail and Recreation | 1 | 0.503 | -0.178 | 0.958 | 0.855 | -0.863 | 0.922 |
| Google:  Grocery and Pharmacy | 0.503 | 1 | 0.01 | 0.6 | 0.66 | -0.622 | 0.457 |
| Google:  Parks | -0.178 | -0.018 | 1 | -0.111 | -0.166 | 0.106 | -0.165 |
| Google:  Transit Stations | 0.958 | -0.6 | -0.111 | 1 | 0.899 | -0.9 | 0.913 |
| Google:  Workplace | 0.855 | 0.66 | -0.166 | 0.899 | 1 | -0.97 | 0.77 |
| Google:  Residential | -0.863 | -0.622 | 0.106 | -0.9 | -0.97 | 1 | -0.788 |
| Apple:  Driving | 0.922 | 0.457 | -0.165 | 0.913 | 0.77 | -0.788 | 1 |

***Table S4.*** *Correlation Matrix for Community Mobility*

| **Coefficient Estimates** | | | |
| --- | --- | --- | --- |
| Variables | 25^th^ Percentile | 50^th^ Percentile | 75^th^ Percentile |
| Constant ($C$) | 0 | 0 | 0 |
| Border Closure ($\beta_{1}^{policcy})$ | 0 | 0 | 0 |
| Initial Business Closure ($\beta_{2}^{policy}$) | -4.644155 | -4.43152 | -4.22604 |
| Educational Facilities Closed ($\beta_{3}^{policy}$) | -11.54166 | -11.1959 | -10.7803 |
| Non-essential Service Closed ($\beta_{4}^{policy}$) | -1.842748 | -1.71596 | -1.58752 |
| Stay at Home Order $(\beta_{5}^{policy}$) | -2.548597 | -2.22569 | -1.88736 |
| Contact Restriction $(\beta_{6}^{policy}$) | -16.99543 | -16.6377 | -16.2624 |
| Retail outlets Closed $(\beta_{7}^{policy}$) | -4.559506 | -4.43518 | -4.31558 |
| Google Trends $(\beta^{trend}$) | -4.878719 | -4.78912 | -4.71101 |
| Tmax $(\beta^{temp}$) | 0.070563 | 0.081985 | 0.092904 |
| Baden-wurttemberg ($state_{1}$) | -0.395887 | -0.28022 | -0.14904 |
| Bayern ($state_{2}$) | -3.119428 | -2.99275 | -2.82677 |
| Berlin ($state_{3}$) | -5.464731 | -5.27469 | -5.11732 |
| Brandenburg ($state_{4}$) | 5.239694 | 5.425942 | 5.595277 |
| Bremen ($state_{5}$) | -1.799361 | -1.6243 | -1.42398 |
| Hamburg ($state_{6}$) | -7.498475 | -7.344 | -7.18164 |
| Hessen ($state_{7}$) | 0 | 0 | 0 |
| Mecklenburg-Vorpommern ($state_{8}$) | 0 | 0 | 0 |
| Niedersachsen ($state_{9}$) | -1.002236 | -0.86896 | -0.72117 |
| Nordrhein-Westfalen ($state_{10}$) | 0 | 0 | 0 |
| Rheinland-Pfalz ($state_{11}$) | 0.1722964 | 0.320479 | 0.456829 |
| Saarland ($state_{12}$) | -2.159837 | -2.03661 | -1.90153 |
| Sachsen ($state_{13}$) | 0 | 0 | 0 |
| Sachsen-anhalt ($state_{14}$) | 2.5614361 | 2.731737 | 2.880114 |
| Schleswig-holstein ($state_{15}$) | 1.3204712 | 1.470629 | 1.625935 |
| Week 0 ($week_{o})$ | 0 | 0 | 0 |
| Week 1 ($week_{1})$ | 4.0150372 | 4.184664 | 4.360424 |
| Week 2 ($week_{2})$ | 6.5606947 | 6.709731 | 6.863293 |
| Week 3 ($week_{3})$ | 2.0989409 | 2.313502 | 2.560254 |
| Week 4 ($week_{4})$ | -1.081132 | -0.76835 | -0.42437 |
| Week 5 ($week_{5})$ | 0.4573945 | 0.644298 | 0.871296 |
| Week 6 ($week_{6})$ | 3.4641791 | 3.616409 | 3.774666 |
| Week 7 ($week_{7})$ | 0 | 0 | 0 |
| Tuesday ($day_{1}$) | 0.3618764 | 0.477806 | 0.581058 |
| Wednesday ($day_{2})$ | 0 | 0 | 0 |
| Thursday ($day_{3})$ | -0.007188 | 0 | 0 |
| Friday ($day_{4})$ | -0.609046 | -0.5159 | -0.41635 |
| Saturday ($day_{5})$ | -1.055083 | -0.9451 | -0.82874 |
| Sunday ($day_{6})$ | 0 | 0 | 0 |

***Table S5.*** *Parameter Estimates from Lasso Regression Model*

**References**

(1) Li R, et al. Substantial undocumented infection facilitates the rapid dissemination of novel coronavirus (SARS-CoV2). Science. 2020. DOI:10.1126/science.abb3221

(2) Robert Koch Institute “Current Situation Report of the RKI to COVID-19" <https://www.rki.de/DE/Content/InfAZ/N/Neuartiges_Coronavirus/Situationsberichte/Gesamt.html> Accessed: May 9, 2020

(3) He, X., Lau, E.H.Y., Wu, P. *et al.* Temporal dynamics in viral shedding and transmissibility of COVID-19. *Nat Med* **26,**672–675 (2020). https://doi.org/10.1038/s41591-020-0869-5

(4) J. T. Wu, K. Leung, G. M. Leung, Nowcasting and forecasting the potential domestic and international spread of the 2019-nCoV outbreak originating in Wuhan, China: A modelling study. *Lancet* **395**, 689–697 (2020). DOI:10.1016/S0140-6736(20)30260-9 Medline

(5) Lauer, Stephen A. et al. The Incubation Period of Coronavirus Disease 2019 (COVID-19) From Publicly Reported Confirmed Cases: Estimation and Application. *Annals of Internal Medicine*, 172(9), 577-582 (2020). doi: 10.7326/M20-0504

(6) Li, Qun et al. Early Transmission Dynamics in Wuhan, China, of Novel Coronavirus–Infected Pneumonia. *New England Journal of Medicine*, 382, 1199-1207 (2020)
doi: 10.1056/NEJMoa2001316.

(7) Diekmann, O., Heesterbeek, J.A.P. & Metz, J.A.J. On the definition and the computation of the basic reproduction ratio *R* _0_ in models for infectious diseases in heterogeneous populations. *J. Math. Biol.* **28,**365–382 (1990). https://doi.org/10.1007/BF00178324

(8) Van Den Driessche P., Watmough J. Reproduction numbers and sub-threshold endemic equilibria for compartmental models of disease transmission. Mathematical Biosciences (2002) 180:29–48.

[9] Bryan E Dowd, William H Greene, and Edward C Norton, Health Serv Res. (2014) Apr; 49(2): 731–750.

[10] Apple Community Mobility Reports: <https://covid19.apple.com/mobility> Accessed: July 21, 2020

[11] Deadorf, Ariel. "Tableau (version 9.1)." Journal of the Medical Library Association, 104 (2), 2016, p. 182+. Accessed 21 Sept. 2020.
